# UK guidelines for the management of soft tissue sarcomas

**DOI:** 10.1038/s41416-024-02674-y

**Published:** 2024-05-11

**Authors:** Andrew J. Hayes, Ioanna F. Nixon, Dirk C. Strauss, Beatrice M. Seddon, Anant Desai, Charlotte Benson, Ian R. Judson, Adam Dangoor

**Affiliations:** 1 The Sarcoma Unit, The Royal Marsden NHS Foundation Trust, London, SW3 6JJ UK; 2 The Institute of Cancer Research, London, SM2 5NG UK; 3 Department of Clinical Oncology, The Beatson West of Scotland Cancer Center, Glasgow, G12 0YN UK; 4 Department of Medical Oncology, University College London Hospital NHS Foundation Trust, London, NW1 2BU UK; 5 The Midlands Abdominal and Retroperitoneal Sarcoma Unit, Queen Elizabeth Hospital, Birmingham, B15 2WB UK; 6 Department of Medical Oncology, University Hospitals Bristol & Weston NHS Foundation Trust, Bristol, BS1 3NU UK

**Keywords:** Sarcoma, Chemotherapy, Surgical oncology, Radiotherapy, Cancer imaging

## Abstract

Soft tissue sarcomas (STS) are rare tumours arising in mesenchymal tissues and can occur almost anywhere in the body. Their rarity, and the heterogeneity of subtype and location, means that developing evidence-based guidelines is complicated by the limitations of the data available. This makes it more important that STS are managed by expert multidisciplinary teams, to ensure consistent and optimal treatment, recruitment to clinical trials, and the ongoing accumulation of further data and knowledge. The development of appropriate guidance, by an experienced panel referring to the evidence available, is therefore a useful foundation on which to build progress in the field. These guidelines are an update of the previous versions published in 2010 and 2016 [1, 2]. The original guidelines were drawn up by a panel of UK sarcoma specialists convened under the auspices of the British Sarcoma Group (BSG) and were intended to provide a framework for the multidisciplinary care of patients with soft tissue sarcomas. This iteration of the guidance, as well as updating the general multidisciplinary management of soft tissue sarcoma, includes specific sections relating to the management of sarcomas at defined anatomical sites: gynaecological sarcomas, retroperitoneal sarcomas, breast sarcomas, and skin sarcomas. These are generally managed collaboratively by site specific multidisciplinary teams linked to the regional sarcoma specialist team, as stipulated in the recently published sarcoma service specification [3]. In the UK, any patient with a suspected soft tissue sarcoma should be referred to a specialist regional soft tissues sarcoma service, to be managed by a specialist sarcoma multidisciplinary team. Once the diagnosis has been confirmed using appropriate imaging and a tissue biopsy, the main modality of management is usually surgical excision performed by a specialist surgeon, combined with pre- or post-operative radiotherapy for tumours at higher risk for local recurrence. Systemic anti-cancer therapy (SACT) may be utilised in cases where the histological subtype is considered more sensitive to systemic treatment. Regular follow-up is recommended to assess local control, development of metastatic disease, and any late effects of treatment.

## Introduction

### Rationale and objective of guidelines

Soft tissue sarcomas (STS) are a relatively uncommon group of malignancies. On average a general practitioner may only see one sarcoma in their career. To improve diagnosis and treatment of these tumours, management was rationalised to peer-reviewed regional soft-tissue sarcoma services. An outline of best practice was set out in the National Institute for Health and Clinical Excellence Improving Outcomes Guidance for people with sarcoma [4] published in 2006 and subsequent quality standards for benchmarking published in 2015 [5].

These guidelines review current evidence concerning management of soft-tissue sarcoma and provide recommendations to support best practice. They are not intended to be prescriptive but aim to improve the quality of care for patients with STS by helping identify and inform the key decisions involved in their management. They will hopefully provide a useful resource for sarcoma services to help guide multidisciplinary team (MDT) case discussions, and patient management.

### Methods

This updated guideline has been authored and reviewed by specialists from the British Sarcoma Group involved in diagnosing and treating patients with sarcoma. As with previous versions, the current NICE (National Institute for Clinical Excellence), ESMO (European Society for Medical Oncology) and NCCN (National Comprehensive Cancer Network) guidance documents have been reviewed, tailoring the recommendations for UK practice. It provides a summary of the current state of established knowledge in sarcoma diagnosis and management, with guidance on what is considered current best practice in the UK. It has been derived by a consensus of expert opinion based on their interpretation of currently available data, and their own clinical experience. Levels of evidence and grades of recommendation (Table 1) have been provided (where appropriate) for all of the key recommendations.

**Table 1:** Definition of ‘Levels of evidence’ and ‘Grades of recommendation’.

Levels of evidence
I Evidence from at least one large randomised, controlled trial of good methodological quality (low potential for a bias) or meta-analyses of well-conducted randomised trials without heterogeneity
II Small randomised trials or large randomised trials with a suspicion of bias (lower methodological quality) or meta-analyses of such trials or of trials with demonstrated heterogeneity
III Prospective cohort studies
IV Retrospective cohort studies or case–control studies
V Studies without control group, case reports, and experts’ opinions
Grades of recommendation
A Strong evidence for efficacy with a substantial clinical benefit, strongly recommended
B Strong or moderate evidence for efficacy but with a limited clinical benefit, generally recommended
C Insufficient evidence for efficacy or benefit does not outweigh the risk or the disadvantages (adverse events, costs, …), optional
D Moderate evidence against efficacy or for adverse outcome, generally not recommended
E Strong evidence against efficacy or for adverse outcome, never recommended

### Scope of guidelines

These recommendations apply principally to soft tissue sarcomas arising from limbs and trunk and although, where appropriate, specific guidance is given according to histological subtype it is recognised that some tumours, for example, Ewing sarcoma, and rhabdomyosarcoma, require a different approach to management, and are excluded from this guidance [6]. These rare subtypes are relatively more common in paediatric and young adult patients. Ewing sarcoma arising in soft tissue are managed in accordance with guidelines for Ewing sarcoma of bone whilst rhabdomyosarcoma treatment is commonly guided by International Clinical Trial protocols such as FaR-RMS (https://www.birmingham.ac.uk/research/crctu/trials/far-rms/index.aspx). For other histologies, similar to adult-type soft tissue sarcomas, arising in children and young people (often referred to as non-rhabdomyosarcoma, soft tissue sarcomas, NRSTS) much less evidence exists for optimal management, in particular the application of chemotherapy and radiotherapy. However, management is likely to be similar in all age groups and so close working between children’s cancer MDTs and sarcoma MDTs should be regarded as best practice.

Specific recommendations on the management of sarcomas arising at defined anatomical sites (retroperitoneal sarcomas, gynaecological sarcomas, breast sarcomas, skin sarcomas) and certain borderline tumours, which are often referred to sarcoma MDTs, are made within these guidelines. Bone sarcomas and gastrointestinal stromal tumours (GISTs) are subject to their own specific BSG guidelines.

### Specialised soft-tissue sarcoma services

The UK National Sarcoma Service specification [3] stipulates that each specialist sarcoma service must have a multidisciplinary team (MDT) made up of radiologists, surgeons, medical and clinical oncologists, pathologists, specialist nurses, and an MDT co-ordinator. The surgical team will comprise at least two dedicated sarcoma specialist surgeons who will be core members of the MDT [4]. Because of the variety of anatomical locations of STS there is a requirement for extended organ-based surgical specialist input to the sarcoma MDT and collaborative working with other MDTs that provide site-specific skills (e.g. gynaecological sarcomas, urological sarcomas, head and neck sarcomas). Guidance for the collaborative relationships between sarcoma and other MDTs is described in the service specification [3].

The MDT will hold weekly meetings to discuss all new cases of sarcoma, plus patients with a high diagnostic suspicion of sarcoma, plus some patients on treatment. The MDT meeting outcomes should be clear and provided promptly to referring clinicians. To deal with large volumes of referrals, centres may use optimised pathways, and separate, smaller, diagnostic MDTs to ensure that the full MDT meetings remain efficient and manageable.

## Epidemiology

Sarcomas comprise a heterogeneous group of approximately 80 entities defined by the 2020 World Health Organization (WHO) classification based on a combination of distinctive morphological, immunohistochemical and molecular features, often with a distinct age distribution, site of presentation, natural biological behaviour, and prognosis [7].

Historically, because of the heterogeneity of this group of tumours, the true incidence has been under-reported. In the UK there are ~5300 new diagnoses of sarcoma per annum (including soft tissue sarcoma, bone sarcoma and gastrointestinal stromal tumours). Sarcoma diagnoses now makeup about 1.4% of all cancer diagnoses in the UK [8]. Most are soft tissue sarcomas with 4295 new cases annually in England in 2017-19, whilst in 2016–18 there were 554 cases of bone sarcoma diagnosed per year [9].

Soft tissue sarcomas may occur at any age, most often in middle aged and older adults; however, as a proportion of paediatric malignancies they are relatively common comprising 7–10% of all childhood cancers. They are an important cause of death in the 14–29 years’ age group [10–12].

Sarcoma survival rates have been very gradually increasing over the last two decades in the UK and are influenced by patient age, tumour subtype, size, and grade. The five-year overall survival rate for all sarcoma grouped together is 55% [13], although individualised risk assessment is better obtained through widely available nomograms [14].

## Aetiology

For most soft-tissue sarcomas, the aetiology is unknown. There are strong associations with certain inherited genetic conditions, such as a 10% lifetime risk of malignant peripheral nerve sheath tumour (MPNST) in individuals with familial neurofibromatosis, caused by mutations in the *NF1* gene [15, 16]. There is an increased risk of sarcomas, both bone and soft tissue, in patients who have had a familial retinoblastoma, caused by inherited mutations in the *RB* gene [17]. Similarly, there is an increased risk of sarcomas, and other cancers in families with Li-Fraumeni syndrome who have inherited mutations in the *TP53* tumour suppressor gene [18]. The International Sarcoma Kindred study also identified a previously unrecognised polygenic influence in the aetiology of sarcoma that has little concordance with previously defined familial cancer syndromes [19]. Patients and families with these cancer predisposition syndromes are ideally referred to local genetic services for advice, and their general practitioners need to be aware of their higher-than-average cancer risk.

Therapeutic irradiation is the most important environmental factor predisposing to sarcoma, often many years after radiotherapy. It is associated with a number or sarcoma subtypes, particularly angiosarcoma after therapeutic breast irradiation [20], but also most commonly with undifferentiated pleomorphic sarcoma (UPS), angiosarcoma and leiomyosarcoma [21]. When matched for standard prognostic factors (age, size and grade), radiation-induced sarcomas appear to have a worse outcome than sporadic sarcomas of similar subtypes [22]. Chronic congenital or iatrogenic lymphoedema has been associated with cutaneous angiosarcoma, otherwise known as Stewart-Treves syndrome [23]. Cutaneous angiosarcomas and atypical fibroxanthoma (AFX)/pleomorphic dermal sarcomas (PDS) are more common in sun-exposed areas due to UV radiation.

## Clinical presentation

The most common presentation of a sarcoma is a painless enlarging soft tissue mass. The clinical recognition especially if deep-seated (i.e. thigh, retroperitoneum) can be problematic and the median size at diagnosis remains large, at over 9 cm [24]. Because soft tissue sarcomas are rare, can occur at any anatomical site, and have diverse histological types, the clinical recognition of a sarcoma can be difficult and late presentation remains a problem. Clinical criteria (soft tissue mass increasing in size, size >5 cm, deep site or pain) for direct referral from primary care to sarcoma diagnostic services were incorporated into the original National Institute of Clinical Excellence (NICE) Improving Outcomes Guidance for Sarcoma [4]. These clinical criteria however failed to discriminate from much more common benign abnormalities, typically lipomas or cysts, and despite a large increase in numbers of referrals from primary care directly to specialist sarcoma units, the percentage of patients who prove to have sarcoma after direct referral from primary care remains low.

Based on convincing data on the effectiveness of ultrasound for discriminating benign from malignant soft tissue masses [25] NICE produced updated guidelines in 2015 [26] for primary care for early diagnosis of soft tissue sarcomas which were:

- Consider an urgent direct access ultrasound scan (to be performed within 2 weeks) to assess for soft tissue sarcoma in adults with an unexplained lump that is increasing in size.
- Consider a suspected cancer pathway referral (for an appointment within 2 weeks) for adults if they have ultrasound findings that are suggestive of soft tissue sarcoma or if ultrasound findings are uncertain and clinical concern persists.

By far the most common soft tissue mass of the limbs and torso seen in primary care is benign lipoma. Atypical lipomatous tumours (well-differentiated liposarcomas) are manyfold less common and tend to be larger, deep-seated and in the lower limb [27]. In an attempt to increase efficiency of the diagnostic pathway the British Sarcoma Group has produced further guidance to help primary care practitioners and ultrasonographers identify which lipomatous masses identified on ultrasound need referral to sarcoma unit, and those which can be managed by local services [28].

Any retroperitoneal or intra-abdominal mass with imaging appearances on CT or MRI suggestive of a soft tissue sarcoma should be referred to a specialist sarcoma MDT before surgical treatment.

## Referral and assessment

### Regional diagnostic services

Each regional sarcoma services should support the development of efficient pathways for the investigation of suspected sarcomas. This may include providing information to local primary care or radiology services on the initial investigation and onward referral of patients with soft-tissue masses, and effective pathways to make direct suspected-cancer referrals when required. This may well involve the development of local diagnostic services that link to a central treatment centre in a ‘hub and spoke’ model.

### Imaging—diagnostic

Ultrasound provides an effective initial triage tool, and in certain conditions such as soft tissue arterio-venous malformations, may demonstrate pathognomonic features. The majority of soft-tissue lumps are likely to be diagnosed as benign lipomas and the patients can be safely reassured. Ultrasound assessment is however highly user dependent, and therefore in the case of diagnostic uncertainty, an MRI of the affected region should be performed. For soft-tissue tumours affecting the extremity, trunk, and pelvis an MRI provides the most accurate information for diagnosis and surgical/ radiotherapy planning. Plain X-ray may be used to identify bone involvement and risk of fracture, or to detect calcification. For retroperitoneal tumours and intra-thoracic sarcomas, CT is preferred for diagnosis and managing treatment. It is more convenient and provides complete staging information on the same scan.

### Imaging—patient staging

Soft-tissue sarcomas have a predominant pattern of metastases to the lungs and therefore a plain chest X-Ray can serve as a useful initial staging investigation. In the case of sarcoma subtypes with very low or negligible metastatic risk (e.g. atypical lipomatous tumours, classic dermatofibrosarcoma protuberans, small AFX/PDS) or in frail and elderly patients in which the identification of very small volume systemic disease would have no treatment implications, a chest X-ray may be sufficient staging. However, most patients with a confirmed STS, and all those with intermediate and high-grade tumours, should be staged with a CT chest to exclude pulmonary metastases prior to definitive treatment. Though isolated visceral metastases from most sarcomas are uncommon [29], at initial staging inclusion of abdomen and pelvis in the CT is usually performed, especially for myxoid liposarcoma and leiomyosarcoma, and for high-grade sarcomas of the lower extremities. Depending on the histological type and other clinical features, further staging assessments may be advised as below [30]:

- Regional lymph node assessment, by ultrasound or cross-sectional imaging for synovial sarcoma, clear cell sarcoma, angiosarcoma, or epithelioid sarcoma, due to a higher risk of nodal involvement.
- Atypical lipomatous tumours (ALT) of the extremities without evidence of de-differentiation have an extremely low risk of metastatic spread [7] and therefore a chest X-ray may be considered adequate staging.
- In cases of myxoid liposarcoma, soft-tissue metastases are more common and so abdominal and pelvic CT scan should be performed routinely. Alternatively, whole body MRI can also be considered [31].
- Contrast CT or preferably MRI, of the brain should be considered in cases of alveolar soft part sarcoma and clear cell sarcoma due to a higher incidence of brain metastases.
- Positron emission tomography (PET-CT) scanning is not yet proven as a routine investigation in sarcoma although may be considered before performing radical surgery, such as amputation for primary or recurrent disease [32]. It also provides a single investigation which can replace a separate staging CT and bone scan, and is becoming standard in sarcomas of younger patients such as Ewing sarcoma and rhabdomyosarcoma [33, 34]. PET-CT has some potential diagnostic utility in neurofibromatosis 1 (NF1) to identify possible malignant peripheral nerve sheath tumours (MPNST) [35, 36].

### Biopsy

The standard approach to establish a histopathological diagnosis of a suspicious soft tissue mass is by percutaneous core needle biopsy. Multiple cores should be taken to maximise diagnostic yield. This is usually performed under image guidance by a radiologist, but for large clinically obvious masses, core biopsies may be undertaken under clinical guidance by clinicians with suitable experience. The biopsy should be planned in such a way that the biopsy tract can be safely removed at the time of definitive surgery. The risk of seeding a metastasis in a biopsy tract is very small [37], and while the placement of a biopsy site within an area of skin that will be excised is still viewed as ‘good surgical practice’, this consideration should not undermine the importance of gaining a pre-treatment histological diagnosis by core biopsy. It would only be in exceptional circumstances that an incision biopsy would be necessary to gain adequate tissue for a pre-treatment diagnosis in a suspected sarcoma and should only be considered after discussion in a sarcoma specialist unit. A planned excision biopsy with minimal or no surgical margins may be the most practical option for small subcutaneous lesions that are indeterminate on imaging (<2 cm diameter), as such lesions usually prove to be benign. In the rare case that an excision biopsy identified a very small sarcoma, a further wide excision of the surgical bed can then be undertaken. Fine needle aspiration is not recommended as a primary diagnostic modality, although it may be considered for confirming disease recurrence, or nodal metastases.

If biopsy of a lesion is planned of a mass which radiologically appears highly likely to be a sarcoma, then in specialist centres in England additional biopsy cores may be taken. This is so that fresh tissue can be snap frozen for whole genome sequencing (WGS) if the mass is confirmed as sarcoma. Currently this testing is supported by the NHS in England for patients with sarcoma [38, 39]. Blood will also be taken to test for germ-line genetic variants. Results of this testing will only be linked to patient demographic data, and the patients informed of results, if they have been consented appropriately.

### Histology—diagnosis

Histological diagnosis should be made according to the 2020 WHO Classification of Soft tissue and bone to determine the grade and stage of the tumour [7] A soft tissue specialist pathology review of diagnostic biopsies performed outside sarcoma centres is recommended as discrepancy rates between diagnosis made outside specialist sarcoma centres after review by specialist sarcoma pathologist is considerable (ranging from 8 to 11% for major discordance, and 16–35% for minor discordance) [40, 41]. Tumour grade should be provided in all cases where possible based on a recognised system. In Europe, the Fédération Nationale des Centres de Lutte Contre le Cancer (FNCLCC) grading system is generally used, which distinguishes three malignancy grades based on differentiation, necrosis, and mitotic rate [42, 43]. Because of tumour heterogeneity and the underrepresentation of necrosis in small samples a core biopsy may underestimate tumour grade compared with final pathology [44]. Additional information may be provided by radiological imaging, and histology may be modified following assessment of the complete surgical resection specimen. Some histologic subtypes cannot be grade using the FNLCC system, such as myxoid liposarcoma, in which case type-specific rules apply.

Pathologic diagnosis relies on morphology and immunohistochemistry. Increasingly it should be complemented by ancillary molecular diagnostic modalities including fluorescent in-situ hybridisation, and reverse transcription polymerase chain reaction (RT-PCR). Increasingly these techniques are being replaced by next generation sequencing (NGS) to confirm pathognomonic genetic markers refining diagnoses and identify novel alterations. Sarcoma biopsy samples can be sent for NGS to identify both genetic variants to support accurate diagnosis but also potentially to help guide treatments. Chromosomal translocations resulting in pathogenic gene fusions are important in a large proportion of sarcomas and may be used to guide therapy. For example the identification of an NTRK fusion, usually ETV6-NTRK3 will permit treatment with a TRK inhibitor [45].

### Staging of soft tissue sarcomas

The most widely used staging system for soft tissue sarcoma is produced by the American Joint Committee on Cancer (AJCC) which stages patients based on the size of the tumour, histopathological grade using the FNCLCC grading system, the presence or absence of nodal and/or distant metastases. The Stage groups I–III comprise localised disease carrying incrementally greater metastatic risk; Stage IV indicating metastatic disease. The major change in the 8th edition of the AJCC staging system is the subdivision of the size criteria (T stage) into four groups compared with two previously, the inclusion of nodal metastasis into stage IV disease, and the inclusion of additional detail on tumours arising at specific anatomical sites (such as the head and neck) and of certain histological subtypes (e.g. gastro-intestinal stromal tumours) [46].

There are also several sarcoma nomograms based on consolidated data from large international patient registries with validation cohorts that complement and build upon the AJCC staging system [46], some of which have been developed into online prediction tools [14, 47, 48]. These allow for the individualised risk assessment of disease free and overall survival based on patient age, completeness of resection, anatomical site, and histological grade and subtypes that are not included in the AJCC staging system.

**Table:** 

Key recommendations: clinical presentation, referral and assessment:
1) Any patient with an unexplained lump that is increasing in size, should be considered for a direct access ultrasound scan to be performed within 2 weeks.
2) Consider a suspected cancer pathway referral for adults if they have ultrasound scan findings that are suggestive of soft tissue sarcoma or if ultrasound findings are uncertain and clinical concern persists.
3) Any retroperitoneal or intra-abdominal mass with imaging appearances suggestive of a soft tissue sarcoma should be referred to a specialist MDT before biopsy or surgical treatment.
4) All patients with a suspected STS should be managed by a specialist Sarcoma MDT as specified in the NICE guidance
5) A pre-treatment histopathological diagnosis should be made, if possible, by percutaneous core biopsy, which should be reviewed by a specialist sarcoma pathologist for diagnostic confirmation, and appropriate molecular and genomic analysis.
6) Cross-sectional imaging of the primary tumour, usually in the form of magnetic resonance imaging (MRI) is recommended prior to definitive surgery.
7) Imaging of the thorax by CT scan for lung metastases should be performed prior to radical treatment. Further staging may be considered depending on subtype and location of the sarcoma.

## Management of localised disease

All patients should have their care managed by a formally constituted sarcoma MDT. Decisions about surgery, chemotherapy, radiotherapy, and the timing of all these modalities should be made by the Sarcoma MDT. For site-specific STS (e.g., gynaecological, head and neck) there should be a formal relationship between the sarcoma MDT and the site-specific MDT. Coordination with the sarcoma MDT helps to ensure optimal management of the sarcoma subtypes, recruitment to clinical trials, and enhances accurate data collection on sarcoma diagnoses and outcomes. In all cases the treatment options will be discussed with the patient, who should be supported by a specialist nurse.

For most limb and truncal tumours, function-preserving surgery and in selected cases, combined with pre- or postoperative radiotherapy is standard treatment, and achieves high rates of local control whilst maintaining optimal function. Radiotherapy may be avoided in patients with low-grade tumours that have been completely resected, or those with small, superficial high-grade tumours resected with wide margins.

### Surgery

#### Surgery for localised disease

Surgery is the standard treatment for all patients with adult-type, localised soft tissue sarcomas, and should be performed by a surgeon who has appropriate training in the treatment of sarcoma. Evaluation of the resectablity of a tumour is determined by the surgeon in consultation with the Sarcoma MDT, and depends on the tumour stage, the anatomical location, and the patient’s comorbidities. The primary aim of surgery is to completely excise the tumour with a margin of normal tissue. Excisional surgery needs to be guided by the principles of surgical oncology and consider anatomical location of the tumour, the histopathology of the sarcoma, the functional consequences of resection. In certain circumstances this may result in widely clear margins in all surgical dimensions obviating the need for adjuvant radiotherapy. In others circumstance it may be acceptable to leave a close or planned microscopic positive margin off a critical structure, supplemented with neo/adjuvant radiotherapy with low rates of local recurrence [49]. In some rare situations, amputation may still be the most appropriate surgical option to obtain local control and offer the best chance of cure.

Plastic surgical reconstruction is an integral part of limb-conserving surgery in a proportion of sarcomas, both for skin and/or soft tissue defects coverage following surgery. There should be close collaboration between the resectional and reconstructive surgical teams (if not performed as one procedure), and the clinical oncology team as the decision around timing of radiotherapy which may be influenced by reconstructive considerations.

Patients who have undergone inadvertent surgery without a preoperative diagnosis of sarcoma resulting in unplanned positive margins should be fully staged and undergo an MRI of the surgical bed to look for gross residual disease. In the absence of any gross residual disease re-excision of the surgical bed may be advised, if adequate margins can be achieved with acceptable morbidity. However, if further excisional surgery is likely to result in considerable morbidity or is unlikely to achieve complete clearance of the potentially contaminated surgical bed (as may be the case for deep-seated limb sarcomas or retroperitoneal tumours) then re-excisional surgery may be not appropriate and observation or radiotherapy may be alternative strategies. Most inadvertent operations are undertaken for cutaneous or subcutaneous sarcomas and further wide excisional surgery plus or minus radiotherapy will usually maintain long-term local control. There is conflicting data on the prognostic significance of unplanned excisions on local control but there is no doubt that it requires more complex surgery and a greater need for adjuvant radiotherapy in contrast to planned sarcoma operations.

For large high-grade tumours that are viewed as borderline resectable at presentation, consideration should be given to a neoadjuvant strategy that may include preoperative radiotherapy and/or systemic/regional chemotherapy [50] Preoperative radiotherapy has the advantage that in radiosensitive tumours such as myxoid liposarcoma it is highly likely that a borderline resectable tumour will be downsized to facilitate an easier and more successful operation [51]. However, for tumours with less intrinsic radiosensitivity, the potential advantages of downsizing need to be balanced against the risks of progression during radiotherapy. Systemic chemotherapy alone or in combination with pre-operative radiotherapy may be considered in the sub-group of patients with chemo-sensitive sarcomas.

#### Histology—resection

The report on the resected specimen should comply with the recommendations for reporting STS produced by the Royal College of Pathologists [52]. The pathology report should include an appropriate description of tumour depth (in relation to the superficial fascia) and margin status with reference to relevant anatomical structures that may be pertinent to the quality of the surgical margin (e.g., fascia, periosteum) The pathologic assessment of margins should be made in collaboration with the surgeon, and confirmation obtained as to whether the tumour was excised intact. Tumour size and grade should be documented noting that the latter cannot be reliably assessed after pre-operative treatment with radiotherapy or systemic therapy. In this setting, the tumour may be assessed for histological response to treatment although the prognostic implications are not well established, in contrast to their utility in osteosarcoma or Ewing sarcoma of bone.

If feasible, it is recommended that tumour samples should be collected and frozen, for consideration of WGS if not already performed on the biopsy.

#### Classification of margins

Surgery is the standard treatment for all patients with an adult-type, localised soft-tissue sarcoma. It must be carried out by a surgeon specifically trained in the treatment of this disease and performed mostly within a sarcoma treatment centre. The standard surgical procedure is an en bloc excision with tumour free margins. This implies removing the tumour in a single specimen with a rim of normal tissue around it, while preserving limb function as best possible. The minimal free margin to be considered adequate may depend on many factors, including histological subtype, pre/postoperative therapies, and the nature of resistant anatomical barriers, i.e., muscular fascia, vascular adventitia, periosteum, and epineurium.

A surgeon may adopt a radical approach to the removal of easily resectable muscles or soft tissues in certain aspects, and yet the resection specimen may well have focal marginal areas where the tumour abuts a critical structure that is not resected because the functional consequences would be prohibitive (planned close margin). Even so a definitive oncological resection with a planned close or microscopic positive margin off a critical structure, when coupled with adjuvant radiotherapy is still associated with excellent local control even for high grade tumours [49].

In modern surgical oncology, complete compartmental resections [53] or amputations are undertaken only when the size, biology, and anatomical relations of the sarcoma mandate this more extreme surgery. For certain indolent sarcoma pathologies particularly atypical lipomatous tumours (ALT) of the extremity, planned marginal resections are the favoured surgical approach and the local recurrence rates are acceptably low without adjuvant radiotherapy.

The recent dataset from the Royal College of Pathologists [52] focuses on the clearance in millimetres of the closest surgical margin, the type of tissue at the margin (eg. fascia, fat, muscle, or skin), whether the invasive margin is infiltrative or pushing, and presence or otherwise of vascular invasion. It is recognised that there is likely to be wide variation in the use of these descriptions and a more pragmatic approach, used in other cancer types, may be to simply classify the margins according to whether there is tumour at the cut edge or not. The AJCC manual [46] describes an R0 margin as free of malignancy, an R1 margin is defined as microscopic tumour cells present at the inked border of the specimen, and R2 refers to a grossly positive margin. Other authors have defined a surgical margin of < 1 mm from tumour as microscopically positive; this system has likewise been reported as prognostic for local recurrence [54].

Margin assessment is complex and must consider the histological subtype of the resected sarcoma, neoadjuvant treatment and the nature of the R1 resection margin. A microscopic positive resection margin at an intentionally preserved critical structure (planned close margin) may have quite different prognostic significance to a multifocal R1 margin on the muscular surface of a resected specimen [49].

#### Isolated limb perfusion

Isolated limb perfusion (ILP) can be a useful pre-operative technique for reducing the size of difficult, but potentially resectable, tumours in an extremity where limb preservation may not otherwise be possible. ILP employs local high-dose chemotherapy plus tumour necrosis alpha, and hyperthermia, restricted to the affected limb using arterial and venous cannulation and a tourniquet. ILP has been shown to shrink peripheral tumours, thus rendering them operable by marginal resection with excellent local control rates and should be considered in selected cases of locally advanced disease. It is also of particular importance as an adjunct to surgical resection for local recurrence in the post-radiotherapy setting where further radiotherapy cannot be delivered, and close margins are likely. In addition, ILP may be considered for palliation of unresectable sarcomas that would otherwise require an amputation, although if the tumour subsequently remains inoperable and durability of control may be limited [55, 56]. Angiosarcoma of the extremities has a very high complete response rate to ILP, including durable responses [57].

#### Excision of the primary tumour in the presence of metastatic disease

Surgical resection of the primary tumour remains an option as a palliative procedure in patients with metastatic disease. However, radiotherapy or chemotherapy may be more appropriate, and the decision must consider factors such prognosis, symptoms, co-morbidity, the expected morbidity of surgery, histological sub-type and the extent of metastases.

### Radiotherapy

#### Post and pre-operative radiotherapy: indications and dose-fractionation schedules

The addition of radiotherapy to surgery allows preservation of function with similar local control rates and survival, to radical resection (compartmental excision/amputation) [58]. It also reduces the risk of local recurrence compared with surgery alone [59, 60]. Both pre- or post-operative radiotherapy are considered standard approaches for most intermediate or high-grade soft tissue sarcomas [61], although in the UK pre-operative radiotherapy is more often employed.

Many patients with low-grade tumours will not require radiotherapy However, it should be considered for those with large, deep tumours with close or incomplete margins of excision, in whom re-excision is not possible, especially if adjacent to vital structures that could limit further surgery in the future.

Local control is similar for both pre- and post-operative radiotherapy [62]. However, risks of acute and late toxicities differ, with preoperative radiotherapy being associated with increased acute, post-operative complications compared to the standard post-operative treatment [62], and post-operative radiotherapy being associated with increased late toxicity compared with pre-operative radiotherapy [63].

The VORTEX randomised clinical trial of post-operative radiotherapy for extremity soft-tissue sarcomas compared the standard post-operative two-phase, shrinking field radiotherapy technique, with a single phase to a smaller treatment volume. The aim of the study was to potentially spare normal tissue, and hence improve subsequent limb function, without compromising local control [64, 65] Results showed no difference in limb function at two years between the treatment arms, and no evidence that smaller radiotherapy planning margins improved limb function. There was no difference in local recurrence, disease free survival or overall survival, but because of lower patient numbers than expected, researchers were not able to confirm that the research arm was non-inferior for local recurrence, and hence there was not a justification for changing practice to using smaller planning volumes [64].

Intensity-modulated radiation therapy (IMRT) should be considered to optimise dose conformality and target volume coverage, aiming to reduce acute and late toxicity [66]. The UK phase II IMRiS trial of IMRT in limb sarcomas has shown rate of grade 2 or lower soft tissue fibrosis at 2 years after IMRT of 11.8%, which represents a clinically meaningful reduction compared to a 30% historical rate after 3D conformal radiotherapy. Similarly low rates of skin, joint and bone late toxicity, and limb oedema, were observed at 2 years, supporting the effectiveness of IMRT in reducing late radiation toxicity [67].

The recommended dose for post-operative radiotherapy is 60–66 Gy in 1.8–2 Gy fractions, and for pre-operative radiotherapy is 50–50.4 Gy Gy in 1.8–2 Gy fractions [61]. Timing of surgery is ~4–8 weeks after completion of radiotherapy. Existing evidence does not supporting the role for a post-operative boost if resection margins are positive, as this is unlikely to be beneficial and may result in excess late toxicity [68, 69]. In recent years, there has been increasing interest in short course hypo-fractionated schedules for pre-operative radiotherapy delivering 25–30 Gy in five fractions over one week, which appear to achieve equivalent local control rates to longer fractionation schedules, without increased toxicity [70–72].

Decisions between pre-operative and post-operative radiotherapy are made on an individual-patient basis at the MDT, taking multiple patient and tumour factors into consideration. Pre-operative radiotherapy treatment volumes are generally smaller than post-operative volumes because the tumour is in situ which facilitates radiotherapy planning, and radiotherapy margins used are smaller. It may be the best option for patients when the priority is to reduce late radiation toxicity, or when the tumour is or borderline operability and pre-operative radiotherapy might render the tumour operable [50]. In addition, it may be particularly suitable for patients with particularly radiosensitive tumours such as myxoid liposarcoma, where a significant tumour shrinkage can be achieved following radiotherapy, facilitating surgery [50]. Pre-operative radiotherapy may not be suitable for patients with rapidly growing, painful tumours, for whom early surgery and post-operative radiotherapy may be the better option. In addition, as pre-operative radiotherapy is associated with a higher incidence of post-operative morbidity including acute wound healing problems, it may not be suitable for patients, or tumour locations, where wound healing is anticipated to be problematic.

#### Definitive radiotherapy

The use of radiotherapy alone as local therapy is unusual in the treatment of sarcoma. However, in a small number of cases the sarcoma may be considered unresectable due to location, local invasion, or because resection would lead to unacceptable morbidity or a poor functional outcome. In these cases, radiotherapy can sometimes provide durable local control. Outcomes are related to tumour size, grade, and radiotherapy dose [73–75]. Doses of over 60 Gy may be used with recommended dose of 66 Gy in 33 fractions over 6.5 weeks [76].

#### Palliative radiotherapy

Radiotherapy plays an important role in the palliation of symptoms from local or distant metastatic disease. Dose-fractionation is selected on an individual patient basis. A range of dose-fractionation regimes can be applied as appropriate, including: 8 Gy single fraction, 20 Gy in 5 fractions over 1 week, 30 Gy in 10 fractions over 2 weeks, 36 Gy in 12 fractions over 2.5 weeks, 39 Gy in 13 fractions over 2.5 weeks, 40 Gy in 15 fractions over 3 weeks [76].

#### Proton beam therapy

Proton therapy is a highly specialised method of delivering radiotherapy to a target volume, whilst minimising dose to normal tissue beyond it. It can therefore enable delivery of high radiotherapy doses when required, and reduction of late normal tissue toxicity, particularly of importance for children, teenagers, and young adults. It is considered for a number of defined indications in sarcoma patients which include spinal and paraspinal bone and soft-tissue sarcomas in adults to enable optimal dose delivery, and broader indications for children and teenagers to reduce late toxicity [77, 78]. It is commissioned by NHS England where applications for treatment are considered by a ‘Proton Panel’. Proton Therapy is delivered at The Christie NHS Foundation Trust in Manchester, and University College Hospital (UCLH) NHS Foundation Trust [79].

### Chemotherapy

#### Neoadjuvant and adjuvant chemotherapy

The role of neoadjuvant and adjuvant chemotherapy in soft-tissue sarcomas has been difficult to establish definitively, although the data supporting it has built up in recent years. It may be considered for patients with higher-risk tumours, and potentially more chemo-sensitive subtypes, such as myxoid round cell sarcoma, synovial sarcoma, uterine leiomyosarcoma, and desmoplastic small round cell tumour (DSRCT). The lack of clear evidence supporting adjuvant chemotherapy may be partially explained by the heterogeneity in response to chemotherapy that we see in tumours, even if they appear morphologically similar [80]—we do not yet have reliable biomarkers to predict response, and thus potential benefit.

It may be particularly appropriate to consider adjuvant chemotherapy in situations where local relapse would be untreatable or where adequate radiotherapy cannot be administered owing to the sensitivity of adjacent structures, for example the heart or spinal cord. A meta-analysis published in 1997 reported an improvement in local control and progression free survival; however, although there was a trend towards an overall survival benefit this was not statistically significant [81]. These data have been supported by two meta-analyses [82, 83]. The latter did not use original trial data and included a small Italian trial which, when published in 2001, reported a significant survival benefit [84]. The EORTC adjuvant therapy trial 62931 [85], the largest trial of adjuvant chemotherapy for STS, failed to demonstrate any clear benefit from chemotherapy in local control, relapse-free survival or overall survival. However, this study was criticised for the low dose of ifosfamide used and for the inclusion of intermediate-grade patients at low risk of relapse. The data were revisited recently stratifying patients using the Sarculator app based on the nomograms published by Callegaro et al. [86]. Those patients with extremity or trunk-wall sarcoma and a predicted 10-year OS of less than 51% did benefit from adjuvant chemotherapy, with a halving in the risk of recurrence or death (HR 0.46) [87]. This supports the use of adjuvant chemotherapy for those patients at high risk of recurrence and death.

A more modern approach, which can be combined with pre-operative radiotherapy, is administering chemotherapy in the neoadjuvant setting. A randomised clinical trial in high-risk patients with extremity or trunk wall sarcoma has helped to clarify the question. A prospective study of pre-operative histotype-tailored (HT) chemotherapy compared with a combination of anthracycline and ifosfamide (AI), stratified by tumour type failed to show an advantage for HT over AI. However further analysis was performed stratifying patients by risk according to the Sarculator nomogram. High-risk patients were those with predicted 5-year survival of <60%. A survival advantage was demonstrated for 3 cycles of AI compared with HT therapy in high-risk patients, with a 5-year OS of 0.66 in the AI arm, compared with 0.55 for HT. The high-risk patients treated with AI had higher 5-year overall survival rates (0.66) than predicted by the Sarculator nomogram (0.58) suggesting efficacy for the neoadjuvant AI [88]. It is important to note that, to date, no randomised trial has tested the superiority of neoadjuvant CT vs immediate surgery in operable patients.

Both pre-operative chemotherapy and radiotherapy can be offered in appropriate cases. Due to concerns about toxicity from concurrent radiotherapy with anthracyclines, 2 or 3 cycles of AI chemotherapy followed by radiotherapy, potentially concurrently with a further cycle of ifosfamide is sometimes utilised. However, there are data to support safe administration of AI concurrently with radiotherapy at 44–50 Gy [89]. It should be noted that no randomised trial has directly tested whether neoadjuvant chemotherapy is superior to immediate surgery in operable patients, so post-operative adjuvant chemotherapy in high-risk patients remains a valid option to consider, particularly in settings where pre-operative radiotherapy is not planned.

**Table:** 

Key Recommendations: Management of Localised Disease
1) Surgery is the standard treatment for most patients with localised STS (IV,A)
2) For those patients with resectable disease, a wide excision through normal uninvolved tissues is the surgical procedure of choice. With the addition of adjuvant radiotherapy a close, but tumour-free margin (R0) may be adequate.(II,A)
3) Where a wide excision is not possible due to anatomical constraints, a planned marginal or microscopically positive margin against a critical structure, plus radiotherapy, for intermediate and high-grade tumours, may be an appropriate means of achieving tumour control while maintaining physical function. Occasionally, amputation should be undertaken as the only surgical option to achieve adequate margins.(IV,B)
4) For patients with borderline resectable tumours, pre-operative treatment with chemotherapy and/or radiotherapy should be considered depending on histology.(IV,A)
5) Pre- or postoperative radiotherapy is recommended along with surgical resection of the primary tumour for the majority of patients with intermediate and high grade tumours, and for selected patients with large or marginally excised, low-grade tumours. (I,A)
6) The recommended dose for post-operative radiotherapy is 60–66 Gy. The recommended dose for pre-operative radiotherapy is 50 Gy. Pre-operative radiotherapy is advantageous in terms of better long-term functional outcome, with equivalent rates of disease control, when compared with post-operative radiotherapy. There is however an increased risk of acute postoperative wound complications.(I,A)
7) Neoadjuvant or adjuvant chemotherapy is not routinely recommended but should be considered in situations where achieving local control is likely to be compromised, or the prognosis is poor, particularly in more chemo-sensitive sarcoma subtypes. Risk stratification can be performed using nomograms such as Sarculator where patients with 5-year predicted survival < 60% may be most likely to benefit.(II,B)

## Prognosis and follow-up for primary disease

Prognosis following primary treatment can be estimated by well-established nomograms based on sarcoma subtype, grade, depth, size, and patient age [90]; with online calculators available [14, 91]. It appears that outcomes may have improved over the past 20 years. Local recurrence is related to grade, margins of excision, and use of radiotherapy, while the risk for systemic metastasis relate to patient’s age, tumour size, grade, and histological subtype. Whilst most events will arise in the first five years following diagnosis, late relapses may occur, according to this French Sarcoma Group study, particularly in retroperitoneal or very large STS [92].

In common with other tumour sites, there are few published data supporting specific follow-up protocols for STS patients. Follow-up should be discussed with the patient and the rationale and limitations explained. Patients may be reassured by follow-up, and early detection of local relapse or pulmonary metastases may improve prognosis in some patients. However there is an increasing push to Patient-Initiated Follow-up (PIFU) as clinical services struggle with capacity, and empowering patients may provide an efficient and effective strategy in selected cases.

A survey on follow-up illustrated how varied the approach is at different centres, with no agreement on imaging, follow-up intervals, or total duration of follow-up [93]. A reported trial comparing standard follow-up, with greater intensity follow-up, failed to show any difference in outcome [94]. Furthermore, a retrospective study of follow-up for detection of local recurrence, demonstrated that most are detected clinically, casting doubt on the utility of routine surveillance MRI scanning [95].

Recurrence in sarcoma consists most commonly of local relapse, or pulmonary metastases. It is recommended that standard follow-up consists of:

I. clinical history,
II. clinical examination to focus on local recurrence, with imaging MRI or CT when indicated by clinical suspicion, or if the primary site is difficult to examine clinically (e.g. intra-abdominal tumours, or deep-seated tumours beneath a flap),
III. chest X-ray, with subsequent CT chest used for investigating radiographic abnormalities.
IV. Monitoring for late effects of treatment.

In certain cases, this standard follow-up can be extended or adapted according to individual risk or local practice. If a patient were deemed unfit either for pulmonary metastasectomy or systemic treatment, then diagnosing metastases when the patient is asymptomatic has little purpose, for example, the chest X-ray could be dispensed with and referral back to primary care might be most appropriate.

As per the ESMO guidelines [30], it is recommended that patients with intermediate/high grade tumours, which most commonly relapse within 2–3 years, should be followed every 3–4 months in the first 2–3 years, then twice a year up to the fifth year, and once a year thereafter for a minimum of 8 to 10 years. It is recommended that patients with low-grade tumours should be followed up every 6 months for 5 years, then annually thereafter, for at least 10 years. In low-grade sarcoma where the risk of local recurrence is the main reason for follow-up, suitably educated patients, with tumours resected from easily examined regions can be considered for discharge from formal follow-up, with an option to self-refer to the service if any abnormality is identified (Patient-initiated follow-up, PIFU). Also, there is a subgroup of patients with good prognosis sarcomas as defined by size and grade in whom the risk of any relapse after five years is extremely remote and shortened follow-up to 5 years should be considered in these patients. Many centres no longer follow-up atypical lipomatous tumours unless there are concerning features such as large size or more abnormal histological appearance.

A further value of follow-up is to monitor for adverse, late effects of treatment. Patients who have received radiotherapy may be at risk of second malignancies or accelerated atherosclerosis in the radiotherapy field. Following chemotherapy there may be deterioration of renal or cardiac function, and reduced fertility. In women, early menopause may require interventions for issues such as bone health. Investigations for late effects of treatment should be considered, such as full blood count, renal profile, hormone profile, and echocardiography. Patients treated in childhood for paediatric sarcomas may be handed on to adult services, and it is important that suitable follow-up continues. Physical disability is a major feature of the survivorship experience of patients treated for soft tissue sarcoma [96], and follow-up should support the patient in trying to minimise the impact of their treatment. Psychological problems associated with relapse risk, altered body image, or loss of function are also common.

**Table:** 

Key Recommendations: Follow-up for primary disease
1. It is recommended that patients with intermediate or high-grade sarcoma are followed up every 3-4 months for the first 2-3 years, then twice a year for up to 5 years, and annually thereafter for a total of 8 to 10 years.
2. Patients with low-grade sarcoma may be followed up every 6 months for 5 years, then annually. Some patients may be discharged for patient-initiated follow-up (PIFU).
3. Standard follow-up practice should consist of:, Review of any new symptoms reported by the patient, clinical examination to focus on local recurrence, with imaging follow-up where indicated by clinical suspicion: Routine chest X-ray to exclude pulmonary metastases. Monitoring for late effects of treatment.

## Prognosis and treatment of advanced disease

In almost all cases, the treatment intention for metastatic disease is palliation. Approximately 50% of patients with high-grade sarcoma develop distant metastases and eventually die of disseminated disease, with a median survival of approximately 12 months from diagnosis of metastases [97–99]. There are more recent data suggesting that this survival figure may be rather conservative with some improvement in outcomes over time to a median of around 18 months [100, 101].

The management of advanced disease is complex; the approach to palliative treatment depends to some extent on whether or not symptoms are present, and the potential toxicities of treatment. In order to achieve control of symptoms such as pain, or dyspnoea, it is often necessary to achieve some degree of tumour shrinkage. Clearly however in the absence of significant symptoms, disease stabilisation is an equally valid aim, to prolong good quality of life. A consistent finding in studies of soft tissue sarcomas is that overall survival, as in GIST [102, 103], correlates with absence of disease progression, not degree of response.

The treatment of advanced disease may involve a combination of various strategies, often used in a stepwise fashion, particularly for those patients with a prolonged disease course. The options will take into account the disease histology, distribution, volume, plus likely sensitivity to systemic treatment. Along with systemic treatment, surgery and radiotherapy may be considered to target symptomatic metastases or in an attempt to prolong the remission period. Other techniques, such as microwave or radiofrequency ablation, may have a role. Medications for pain or other complications such as bone metastases may be considered. Bisphosphonates or denosumab may be useful in reducing fracture risk or bone pain, based on data from other cancers, although radiotherapy or surgery may also be indicated. In some patients, metastases may behave fairly indolently and periods without active treatment are often appropriate. Other areas to focus on are good supportive care, potentially involving specialist palliative care services, in coordination with primary care.

For a number of patients, particularly those with poor performance status or significant comorbidities, standard supportive care with symptom control alone, is often the most appropriate option. Early involvement of community palliative care teams should be considered in all patients with advanced disease.

### Systemic anti-cancer therapy (SACT) for sarcomas

The development of optimal treatment protocols is hampered by the rarity and heterogeneity of sarcomas. The incidence of many of the individual sub-types of soft tissue sarcoma is too small to permit large-scale prospective randomised controlled trials. Accordingly, data are gathered from a range of studies which include single-site and multisite phase 2 trials, retrospective case series, sub-analyses of trials for which a range of histological subtypes are included and, for the rarer sub-types, individual case reports. More recently genomic analysis through NGS or whole genome sequencing is starting to allow more precise tailoring of treatment, for example in tumours harbouring NTRK fusions, but this is only relevant in a very small proportion of patients.

Treatment can be guided by local or regional algorithms and the BSG plans to publish recommendations on the BSG website (https://britishsarcomagroup.org.uk). There are international references such as the ESMO [30] and NCCN guidelines [104].

Systemic treatment options include chemotherapy, tyrosine kinase inhibitors, biological therapies, and immunotherapy. The published response rates for chemotherapy in STS vary enormously, from 10 to 50% depending on the drugs used, patient selection, and histological subtype. It has been established that good performance status, young age, and absence of liver metastases predict a good response to chemotherapy and improved survival time [98]. It is increasingly understood that response rate is only one measure of treatment efficacy with many of the newer therapies leading to a clinical benefit through disease stabilisation. A differential response to chemotherapy according to histological subtype has been noted, and as knowledge increases it is expected that it will become increasingly possible to individualise treatment. For example; synovial sarcoma, leiomyosarcoma and myxoid liposarcoma are recognised as having higher response rates to chemotherapy. Conversely, alveolar soft part sarcoma, extraskeletal myxoid chondrosarcoma and solitary fibrous tumour are generally regarded as insensitive to chemotherapy, and there are only occasional reports of responses in clear cell sarcoma. However, in the era of targeted therapies merely looking at response rates to standard chemotherapy is starting to be superseded by systemic treatment tailored to the histology or genetics of the individual subtype [105–107].

### Selection of SACT

Treatment of advanced disease may involve other modalities such as radiotherapy or surgery, and so multidisciplinary team review is important. Systemic treatment should ideally be guided by established protocols, preferably shared nationally and internationally. There is potential variability in dosing and administration, particularly in the use of agents such as ifosfamide, and care should be taken to use treatment protocols that maximise benefit whilst ensuring optimal management, and minimisation, of potential toxicities. Techniques such as the use of ambulatory infusions can be used to enhance patient convenience, and free-up valuable inpatient resources [108].

In a cost-constrained health system there is a challenge to fund all active agents, particularly in rare diseases. Some of the treatments considered may fall outside current standard NHS funding, and this will have to be taken into account when discussing the options with patients.

In most cases of metastatic soft-tissue sarcoma the choice of first-line chemotherapy will be between single-agent doxorubicin, or combined doxorubicin and ifosfamide. In a European trial a dose-intense ifosfamide/doxorubicin combination did not improve survival, but did deliver a higher response rate and improved progression-free survival, though at the cost of increased toxicity [99]. This may be an important consideration if the patient is symptomatic due to tumour size, or a reduction in tumour volume might facilitate other treatment options. The performance status of the patient and comorbidities will play an important role in treatment selection particularly in view of potential cardiac toxicity of doxorubicin and renal toxicity seen with ifosfamide. An option in locally advanced, or metastatic leiomyosarcoma where a higher response rate is desired, is the combination of doxorubicin and dacarbazine which, in a multi-centre retrospective study appears, more effective than doxorubicin alone or combined with ifosfamide [109].

Standard second-line treatment is ifosfamide, which is also used first-line where anthracyclines are contraindicated, for example in patients at high risk of cardiac complications, or patients pre-treated with anthracyclines. Clinical trials have indicated a dose-response relationship, and a dose of 9–10 g/m^2^ is recommended [110]. In unselected sarcomas the response rate is in the region of 8%, although higher response rates have been observed with high-dose (>12 g/m^2^) and continuous infusion ifosfamide regimens [111, 112]. Responses may be higher in certain subtypes such as synovial sarcoma, whilst from retrospective data leiomyosarcoma is arguably less responsive and alternative agents may be more appropriate [113]. Ifosfamide is usually given over two to three days as an inpatient, but infusional regimens administering treatment via a pump over two weeks have been utilised [114]. Treatment given in this way is usually better tolerated, but so far is most established in retroperitoneal liposarcoma, and is not yet a standard of care. Renal toxicity of ifosfamide can be significant and close monitoring is required. More rarely neurotoxicity is seen, more often in debilitated patients with low albumin levels.

An alternative second-line option is the combination of gemcitabine and docetaxel. Activity has been demonstrated in soft tissue leiomyosarcoma and other tumour types including UPS [115, 116]. The GeDDiS trial in which this regimen was compared with doxorubicin in the first-line setting for all sarcoma subtypes, showed it to be non-inferior, but more toxic [117]. Gemcitabine/dacarbazine combination therapy is another alternative to gemcitabine/docetaxel which avoids the more significant toxicity of docetaxel. It is more effective than gemcitabine alone, and generally well tolerated [118]. Activity of dacarbazine has been reported against solitary fibrous tumour/haemangiopericytoma [119].

Trabectedin, licensed as second-line treatment for all soft tissue sarcomas, was approved by the European Medicines Agency (EMA) on the basis of a randomised trial comparing two different treatment regimens in patients with predominantly leiomyosarcoma and liposarcoma [120]. A trial in patients with leiomyosarcoma and liposarcoma comparing trabectedin with dacarbazine demonstrated significant superiority for trabectedin resulting in the drug being licensed in the USA [121]. Other tumours, such as synovial sarcoma, and particularly myxoid liposarcoma, may also be sensitive. It appears to be active in sarcomas related to chromosomal translocations [122, 123]. When assessing clinical benefit, it should be noted that a period of disease stabilisation may often occur for some time before response is seen. Trabectedin is currently approved by NICE and treatment can continue until disease progression. It exhibits less haematological toxicity than doxorubicin or ifosfamide, but prescribers need to be aware of rare, but potentially serious rhabdomyolysis, and hepatic toxicity.

Beyond the regimens above, there are no standard chemotherapy options and decisions will be made based on patient fitness, and a balance of likely benefit and toxicities. Consideration of previous clinical benefit from chemotherapy, and more chemo-sensitive subtypes of sarcoma may support further treatment. Below are a number of other SACT options:

- Liposomal doxorubicin: could be considered at any line for vascular intimal sarcomas, angiosarcomas [124], cardiac sarcomas, and patients who have received previous anthracyclines, or have impaired cardiac function [125]. It can be combined with ifosfamide. It also has activity in fibromatosis and Kaposi sarcoma.
- Paclitaxel: May be used as first or second-line treatment of angiosarcomas [126].
- Oral cyclophosphamide and prednisolone: A low-toxicity combination suitable for elderly patients unlikely to tolerate more toxic chemotherapy [127].
- Eribulin: received marketing authorisation from the EMA in 2016 for the treatment of unresectable liposarcoma following prior anthracycline-containing therapy. This followed subgroup analysis of a study comparing eribulin with dacarbazine for previously treated patients with liposarcoma or leiomyosarcoma [128]. The data in other sarcoma types is limited [129] and it is not currently funded in England for use in sarcomas.
- Pazopanib: Has data supporting its use in metastatic STS (not liposarcoma). A placebo-controlled study demonstrated a 3-month improvement in progression-free survival in STS, with no particular superiority in any individual subtype [130, 131]. Of note, activity was also seen in refractory desmoplastic small round cell tumour. This class of VEGFR inhibitor (including sunitinib) has also demonstrated activity in haemangiopericytoma/malignant solitary fibrous tumour [132], which is relatively resistant to chemotherapy (although see dacarbazine below), and in refractory desmoid tumours/fibromatosis [133].
- Imatinib: has demonstrated utility in DFSP (dermatofibrosarcoma protuberans) [134], and tenosynovial giant cell tumours [135].
- NTRK inhibitors have been approved as a tumour-agnostic treatment for malignancies driven by an *NTRK*-fusion, demonstrating high response rates and impressive progression-free survival benefits [136]. NTRK-fusions have a frequency of less than 1% in sarcomas, although are almost pathognomonic in some paediatric tumours such as infantile fibrosarcoma.
- Tazemetostat: an oral EZH2 inhibitor has demonstrated efficacy in epithelioid sarcoma in a series of 62 patients [137].
- Immunotherapy: Although the results from trials of checkpoint inhibitors in sarcoma have been generally disappointing compared to other cancer types, there is encouraging evidence of some efficacy in alveolar soft part sarcoma (ASPS) [138], angiosarcoma [139], UPS [140], and metastatic pleomorphic dermal sarcomas (PDS) [141]. The PD-L1 inhibitor atezolizumab has recently been approved by the FDA in the US for the treatment of alveolar soft part sarcoma, as has pembrolizumab for unresectable or metastatic solid tumours with high tumour mutational burden (TMB-H).

Table 2 summarises the potential role of targeted therapies in histological subtypes that are poorly responsive to chemotherapy. There has recently been a rapid increase in knowledge about sarcoma subtypes and potential treatments, led by the revolution in genomics and immunotherapy. Therefore, the information above will not remain current after publication of this guideline, and oncologists treating sarcoma need to remain alert to new clinical trials and treatments that may benefit their patients. Websites such as clinicaltrials.gov and https://sarcoma.org.uk/clinical-trials-hub/ can be a useful source of information on available trials. A challenge is that not all potentially active agents are funded by the NHS in the UK for the indications described. Funding varies across the devolved nations and is regularly under review. These processes however struggle to keep up with the rapid pace of innovation in treatment, and balances between cost and efficacy have to be made.

**Table 2:** The potential role of targeted therapies in histological subtypes that are poorly responsive to chemotherapy [modified from [220]].

STS subtype	Key characteristics	Drug
Extraskeletal myxoid chondrosarcoma	*EWSR1-NR3A3* gene fusion	Pazopanib [221], sunitinib [222]
Solitary fibrous tumour	Typical, i.e., low aggressiveness	Anti-angiogenic TKI [223]
Desmoid fibromatosis	n/a	Sorafenib [212], pazopanib [224], nirogacestat [214]
Alveolar soft part sarcoma	n/a	Anti-angiogenic TKI [225, 226], atezolizumab [227] or combination of TKI /checkpoint inhibitor [228, 229]
Epithelioid haemangioendothelioma	n/a	mTOR inhibitors, e.g. sirolimus, TKIs [230]
Inflammatory myofibroblastic tumour	n/a	ALK inhibitor [231]
Epithelioid sarcoma	n/a	Tazemetostat [137]
PEComa	n/a	Sirolimus [232], NAB-sirolimus [233]
Undifferentiated pleomorphic sarcoma	n/a	Immune checkpoint inhibitor [140]
Angiosarcoma	Radiation-associated	Checkpoint inhibitor [140]

### Management of local recurrence

Local recurrences are often accompanied by metastatic disease and patients should be fully staged. In the absence of overt metastatic disease every attempt should be made to regain local control by further surgery with adequate margins, and radiotherapy (if not used previously). Amputation may be needed in selected cases.

### Management of lung metastases

Following a diagnosis of lung metastases alternatives to SACT may be considered. The decision regarding metastasectomy should be based on disease-free period following primary surgery, absence of other metastases, number of lesions per lung, tumour growth, and evolution of disease [30]. In the absence of a significant disease-free interval, the CT scan (or PET-CT scan to complete staging) should be repeated at a three-month interval, and if no new lesions have appeared and the disease is operable, surgery is usually recommended. The practice of performing an interval scan and delaying surgery can be difficult to explain to patients, but the risk of immediate surgery is that further multiple metastases appear rapidly, rendering the morbidity of surgery pointless, and potentially delaying systemic treatment. Other approaches can also be considered such as radiofrequency or microwave ablation. More recently stereotactic ablative radiotherapy (SABR), a very targeted form of high-dose hypo-fractionated radiotherapy, has become another potential option. While there are few data from prospective studies reporting survival of STS patients treated surgically for thoracic metastases, and the survival benefit remains unproven, in selected patients long-term survivors are reported (20–40% of all patients undergoing lung surgery) [142].

### Management of extra-pulmonary oligometastases

In most cases extrapulmonary metastases will be treated with systemic treatment. In selected cases surgery, radiofrequency ablation (RFA), cryotherapy, or radiotherapy may be considered for limited metastatic disease to prolong remission or reduce symptoms. Electrochemotherapy (ECT) is technique that may be useful in the management of refractory dermal and subcutaneous metastases in certain tumour sub-types, for example angiosarcoma [143].

### Best supportive care

Supportive and palliative care should always be considered in cases of advanced disease. For many patients, systemic therapy, radiotherapy, or surgery may not be appropriate, and an early and honest conversation about treatment options, potential toxicities and quality of life is important. Involvement of a sarcoma specialist nurse to support the patient through the diagnostic process and discussion of options can be invaluable. Early referral to specialist palliative care services in the community should be considered. Although prognostication can be difficult and inexact, most patients and their families will want some idea of likely outcomes, and this should be explored with them. Discussions concerning end-of-life care preferences may also be appropriate.

**Table:** 

Key Recommendations: Systemic Anti-Cancer Therapy for Sarcomas:
1) Systemic treatments for the majority of advanced STS are not curative; median survival time is 12-18 months. Published chemotherapy response rates vary enormously; from 10–50% depending on the drugs used, patient selection, and tumour grade and histological subtype(I,B). Treatment recommendations should be guided by patient performance status, disease extent, rate of progression, and potential sensitivity to treatment (I,A)
2) Standard first-line treatment is single-agent doxorubicin (I,A).
3) Ifosfamide may be used first-line if anthracyclines are contraindicated and is a standard option for second-line therapy (I,B)
4) Although the combination of doxorubicin and ifosfamide has not been demonstrated to improve survival in comparison to single agent doxorubicin first-line, response rates and progression free survival are higher and it may be considered in individual patients where a response would improve symptoms or facilitate other treatment modalities (II,B)
5) Additional second-line agents include trabectedin, and the combination of gemcitabine/ docetaxel or gemcitabine/dacarbazine. The choice of agent depends on histology, toxicity profile and patient preference (II,B).
6) Increasingly treatments more specific for sarcoma subtypes are being elucidated, such as NTRK inhibitors for tumours harbouring NTRK-fusions, and immunotherapy in subtypes such as alveolar soft part sarcoma (ASPS). For those diseases, such as ASPS, which do not respond to chemotherapy, a targeted therapy should be considered first-line, if a suitable drug is available (III,A).
7) Surgical resection of locally recurrent disease should be considered where feasible. For patients with oligometastatic disease surgery, radiotherapy, or ablative therapies (RFA, SABR, cryotherapy, microwave, ECT) should be considered in individual cases, although there are limited data on survival benefit (III,B)

## Site-specific sarcomas

Given the heterogeneity of sarcoma presentations many patients are managed in collaboration with other site-specific multidisciplinary teams. Close collaboration between Sarcoma MDTs and site-specific MDTs at the point of sarcoma diagnosis is the best means of securing an optimum outcome. The recent National Sarcoma Service Specification [3] emphasises that the surgeon undertaking site-specific sarcoma surgery should ideally be a designated extended member of the sarcoma MDT. Retroperitoneal sarcomas must be managed by surgeons who are either core members or extended members of the sarcoma MDT, as this disease has no equivalent non-sarcoma MDT that routinely undertakes multi-visceral resections. Gastrointestinal stromal tumours (GIST) are usually managed in collaboration with GI surgical services and are discussed in separate BSG guidance.

### Retroperitoneal sarcomas

Retroperitoneal sarcomas include various histological subtypes each with specific behaviour, pattern of recurrence and tumour biology. Surgical management should be regionalised within specialist high-volume retroperitoneal sarcoma MDTs, and surgery performed by surgeons specialised in the management of retroperitoneal sarcoma with experience in multi-visceral extended resections, familiar with different sarcoma subtype behaviour, and knowledge of how to tailor treatment accordingly. Current NHS England Sarcoma Service specification [3] define specialist high-volume retroperitoneal sarcoma units by:

- Regular sarcoma MDT meetings which include anatomic expertise in pathology, imaging and surgery;
- Infrastructure and resource to support major intra-abdominal surgery; and
- A recommended case load including surgical resection of an average of 24 new cases of primary retroperitoneal sarcoma per annum.

Patients with retroperitoneal sarcoma can present to a variety of clinicians with non-specific symptoms and can be incidental findings on imaging. Retroperitoneal sarcomas are rare, however the most common subtypes, liposarcomas (70%) and leiomyosarcomas (15%), have characteristic imaging appearances; in future radiomics may assist with diagnosis [144]. Recognition of abnormal fat in the retroperitoneum is most helpful for the diagnosis of liposarcoma, and solid masses originating from major vessels may indicate the second most common subtype leiomyosarcoma. However, because of the spectrum of benign and malignant pathologies which can occur in the retroperitoneum, biopsy in liaison with a specialised soft tissue sarcoma centre should always be performed. Contrast-enhanced CT of the chest, abdomen, and pelvis is used for staging and in helping to plan surgery. MRI and/or CT-PET can be used for problem cases [145].

Pre-treatment image-guided percutaneous coaxial core needle biopsy for histological diagnosis is strongly recommended, unless the imaging reviewed in a specialist sarcoma MDT is pathognomonic of a liposarcoma and no preoperative treatment is planned. Multiple 14- or 16-gauge cores should be obtained to allow enough tissue for immunohistochemistry and molecular subtyping [146]. MDM2 gene amplification status is considered the gold standard for diagnosis of well-differentiated/dedifferentiated liposarcomas [147–149]. Preoperative core needle biopsy via a transperitoneal approach with a co-axial technique is safe and does not affect oncological outcome. Physicians and patients can be reassured that the benefits of core needle biopsy in diagnosing sarcoma and determining its histologic subtype and grade, far outweigh the risks and the incidence of needle tract seeding is extremely low. Laparotomy and open biopsy, or laparoscopic biopsies of suspected RPS should be avoided [37, 150].

Surgery remains the only curative treatment. The optimal time for surgical resection with curative intent is at primary presentation. Surgical planning should consider patient performance status, biological tumour behaviour, oncological risk, and morbidity associated with surgical extent, Anatomical constraints due to nearby vital organs and structures in the retroperitoneum limit the ability to achieve wide resection margins. Surgery should be performed to achieve a macroscopic complete resection, with a single intact specimen encompassing the tumour and involved contiguous organs, while minimising microscopic positive margins [146].

Retroperitoneal liposarcomas have poorly defined margins and an inherent higher risk for local recurrence. For liposarcomas, available evidence shows an extended surgical approach may improve long-term local control. Surgery to resect the tumour and adjacent viscera, irrespective of involvement and clearing all ipsilateral fat, to minimise microscopic positive margins, should be considered. Resection often necessitates ipsilateral nephrectomy, hemicolectomy, psoas fascia/muscle resection and distal pancreatectomy/splenectomy on the left.

Retroperitoneal leiomyosarcomas have more clearly defined borders, with a low risk for local recurrence after complete resection but have a higher risk for systemic metastasis. Extended resections will not improve oncological outcomes which is dictated by metastatic disease. Surgical strategy should aim for complete resection of the tumour with involved organs and preservation of adjacent uninvolved organs [151–154].

Retroperitoneal solitary fibrous tumours (SFT) exhibit a low risk for local recurrence. Although malignant SFTs exist and can be classified post-operatively, the aim of resection should be complete resection with negative margins while preserving uninvolved organs [155]. The activity of radiotherapy in SFT should be considered in the preoperative planning.

Retroperitoneal UPSs of the psoas muscle are usually separated from the retroperitoneum by the psoas fascia and surgery involves removal of the whole psoas muscle and intact tumour with preservation of uninvolved nerves and vessels. Retroperitoneal malignant peripheral nerve sheath tumours should be resected with negative margins, and usually involve sacrifice of the nerve of origin.

In a randomised prospective trial of preoperative radiotherapy plus surgery versus surgery alone, preoperative radiotherapy did not improve abdominal recurrence-free survival and overall survival for the whole population of patients. Preoperative radiotherapy was well tolerated, and the additional morbidity associated with preoperative radiotherapy was acceptable. Abdominal recurrence free survival was significant improved in the low-intermediate grade liposarcoma subgroup and preoperative radiotherapy should be discussed with this group. There was no benefit in preoperative radiotherapy for patients with leiomyosarcomas and high-grade tumours [156].

Postoperative radiotherapy following complete resection is of limited value and associated with significant toxicities and should only be considered in selected cases with a well-defined area at risk for local recurrence. Preoperative chemotherapy for high-grade dedifferentiated liposarcoma and leiomyosarcomas is currently under investigation in a multi-centre prospective randomised study [157]. Preoperative chemotherapy can be considered for chemo-sensitive subtypes such as synovial sarcomas and borderline resectable leiomyosarcoma. The value of adjuvant chemotherapy is not established and cases at high risk for metastatic disease should be individually discussed. The Sarculator nomogram can be used for prognostication [14].

Surgery for local recurrence should be considered on individualised basis within the expertise of a specialist sarcoma MDT. Prognostic factors to consider include age, histological subtype, tumour grade, multifocality, disease-free interval and previous treatment [158].

The risk and pattern of recurrence following resection of RPS are dictated by histological subtype and tumour grade. Liposarcomas tend to recur locoregionally, while high-grade RPS recur more often systemically. Low-grade liposarcomas may recur late even after 10 years while high-grade RPS mostly recur within 5 years of treatment. Follow-up assessment should include clinical evaluation and cross-sectional imaging. The interval between follow-up is not evidence based but could be shorter initially (3–6 monthly) and annually after 5 years. Cross-sectional imaging may detect asymptomatic recurrences long before symptoms develop due to recurrent disease. The potential benefit of earlier detection of recurrent disease is controversial. An initial period of observation of image-detected recurrences may be appropriate to assess behaviour and the likelihood of benefit of further surgery. Clinical assessment only in asymptomatic patients could be discussed with the patient where significant comorbidities or other factors will prohibit active treatment of asymptomatic recurrent disease detected on imaging.

For many patients with advanced disease or significant comorbidities prohibiting surgery, aggressive therapy may not be appropriate, and good symptomatic management, and palliative care support are required.

**Table:** 

Key Recommendations: Retroperitoneal Sarcomas
1) The optimal management of retroperitoneal sarcoma (RPS) is facilitated by pre-treatment diagnosis and image-guided percutaneous core needle biopsy is strongly recommended. (III,A)
2) Biologic behaviour, response to treatment, and clinical outcomes vary by histological subtype of RPS. The management plan, including extent of resection and neoadjuvant strategies, should be formulated accordingly (III,A)
3) The best chance for curative resection is at the time of primary presentation, with the standard of care being en bloc macroscopically complete resection of the tumour and involved/adjacent organs, performed in high-volume specialist sarcoma centres.(III,A)

### Gynaecologic sarcomas

This group, which forms around 2% of gynaecological cancers, includes uterine leiomyosarcomas (LMS), uterine adenosarcoma, and endometrial stromal sarcomas (ESS), which are subdivided by WHO into endometrial stromal nodule, low-grade endometrial stromal sarcoma, high-grade endometrial stromal sarcoma (HG-ESS), and undifferentiated endometrial sarcoma (UES). Carcinosarcomas are considered as epithelial tumours and should be treated accordingly. Management is shared with gynaecological cancer teams, with discussion of cases by the regional soft-tissue sarcoma MDT (British Gynaecological Society Uterine Cancer Guidelines (https://www.bgcs.org.uk/professionals/guidelines-for-recent-publications/). Advanced disease is usually treated by sarcoma specialist oncologists.

#### Uterine leiomyosarcoma

Uterine LMS, a cancer of the smooth muscle, accounts for 35–40% of all uterine sarcomas; LMS predominantly affects patients aged 50–60 years. Pre-operatively it is difficult to differentiate benign leiomyomas from malignant LM. However, there are a number of red flags which increase the risk of inadvertent dissemination of a LMS during surgery, especially if morcellation is used, such as post-menopausal bleeding, rapid enlargement, failure to respond to oestrogen deprivation in a pre-menopausal woman and certain imaging features, such as increased vascularity. These are detailed and referenced in consent advice from the Royal College of Obstetricians and Gynaecologist [159]. Laparoscopic morcellation is contraindicated for uterine sarcoma due to higher risk of recurrence and metastasis [160–164]. Standard surgical management for non-metastatic disease is total abdominal hysterectomy (TAH), with or without bilateral salpingo-oophorectomy (BSO). Retention of the ovaries can be considered in pre-menopausal women. Routine lymphadenectomy is not required, as lymph node involvement is less than 5%. Adjuvant pelvic radiotherapy for FIGO stage I and II disease is not recommended [165]. Adjuvant pelvic radiotherapy may be considered for selected high-risk cases, for example with serosal breach, parametrial involvement, where local relapse may be reduced, although a survival benefit has not been demonstrated. Adjuvant chemotherapy is not routinely recommended due to lack of supportive evidence [166–171]. Chemotherapy for advanced/metastatic disease is as for STS at other sites, noted above, with doxorubicin or doxorubicin/dacarbazine, as first line treatment. Second-line options include gemcitabine as single agent or in combination with docetaxel [116], or dacarbazine [118], or else trabectedin as a single agent [118, 172]. Trial evidence suggests that combining doxorubicin with trabectedin first line, improves progression-free survival [173], although this combination is not yet supported by NICE. There is retrospective evidence that ifosfamide may be less effective in leiomyosarcoma [113].

Oestrogen receptor (ER) and progesterone receptor expression is seen in ~50% of patients with uterine LMS. Some low and intermediate-grade tumours may be sensitive to oestrogen deprivation, (e.g. using aromatase inhibitors), although data are sparse [174, 175]. It can also be an option for patients not considered fit enough for chemotherapy. It is reasonable to evaluate receptor expression in those with relatively indolent tumours.

The management of uterine adenosarcoma is largely as for leiomyosarcoma.

#### Uterine adenosarcoma

Uterine adenosarcoma is a rare biphasic tumour consisting of a malignant stromal and a benign epithelial component. Sarcomatous overgrowth and myometrial invasion are both poor prognostic factors [176]. For patients with early-stage disease standard surgical treatment is total hysterectomy and bilateral salpingoopherectomy [177]*.* There is no proven role for adjuvant therapy due to a lack of evidence in this rare entity. In patients with metastatic adenosarcoma with a predominantly epithelial component endocrine therapy is a therapeutic option. In those patients with sarcomatous overgrowth (>25% of tumour volume) then systemic treatment along a uterine LMS paradigm is advised. There is some evidence to support the use of trabectedin [178].

#### Low-grade endometrial stromal sarcoma (ESS)

This is the second most prevalent gynaecologic sarcoma which is low grade and characterised by an indolent disease course. There is a high incidence of ER and PR expression, and evidence that these tumours respond to hormonal manipulation. Standard surgical treatment is TAH, usually with BSO in pre-menopausal women; hormone replacement therapy (HRT) is contraindicated [179]. There is no routine role for adjuvant hormonal treatment due to lack of evidence to support this. The role of adjuvant pelvic radiotherapy is uncertain given the paucity of published data. Recurrent or metastatic disease may respond to anti-oestrogen therapy, with an aromatase inhibitor, or a progestogen. Tamoxifen is not recommended since its action may be pro-oestrogenic in this setting. Chemotherapy is an option if hormonal therapy fails. Given the indolent nature of the condition, surgery or other local ablative approaches for metastatic disease should be considered.

#### High-grade ESS and undifferentiated endometrial sarcoma

High-grade ESS characterised by *YWHAE-FAM22* transcript, and UES are highly aggressive tumours that do not express ER and PR, with a poor prognosis and uncertain response to systemic treatment [180]. Surgical management is TAH with or without BSO, and the option of adjuvant pelvic radiotherapy [165]. Follow-up protocols and systemic treatment for advanced disease parallel those for adult-type soft tissue sarcomas [30].

**Table:** 

Key Recommendations : Gynaecologic Sarcomas
1) Standard treatment for all localised uterine sarcomas is total abdominal hysterectomy. Lymphadenectomy is not routinely indicated.(III,B)
2) Total abdominal hysterectomy with bilateral oophorectomy is indicated for endometrial stromal sarcoma. These patients should not have post-operative hormone replacement therapy. Use of adjuvant oestrogen deprivation therapy is not indicated.(III,B)
3) Adjuvant pelvic radiotherapy has not been shown to improve survival and is not routinely indicated in FIGO stage I and II disease. However, it could be considered for selected high-risk cases.(IV,C)
4) Advanced/metastatic LMS and undifferentiated endometrial sarcoma are treated with the same drugs as STS at other sites. (IIIB) There is retrospective evidence that ifosfamide may be less effective in leiomyosarcoma (III,C).
5) Advanced/metastatic ESS can be treated with oestrogen deprivation therapy, with an aromatase inhibitor or progestogen. Tamoxifen is not recommended since its action may be pro-oestrogenic (III,C).

### Breast sarcomas

Breast sarcomas comprise one of three principal pathologies, primary sarcomas of the breast, malignant phyllodes sarcoma and radiation induced breast sarcoma [181]. Breast tumours demonstrating sarcomatous differentiation within a metaplastic carcinoma may often be referred to a sarcoma MDT, but these cancers should be managed as epithelial breast tumours, being often managed as triple-negative breast cancers.

The management principles for primary breast sarcoma differ from epithelial breast malignancy in that there is no requirement for axillary staging by sentinel node biopsy, and adjuvant or neoadjuvant chemotherapy is not routinely given for sarcoma of the breast [182]. Due to the variation in clinical practice across the UK and improved clinical outcomes in specialist sarcoma centres, it is recommended that breast sarcomas and phyllodes tumours, of borderline and malignant subtypes, are referred to specialist sarcoma centres for pathology review and MDT discussion. The Association of Breast surgeons (https://associationofbreastsurgery.org.uk) has produced a phyllodes guideline.

Standard surgical treatment remains wide excision with clear margins and either breast conservative surgery (BCS) or mastectomy can be undertaken [183]. For large malignant phyllodes tumours, breast conservation may not be possible. The role of immediate reconstruction needs careful discussion on an individual case basis, as patients with large high-grade tumours are likely to receive postoperative chest wall radiotherapy and carry a significant chance of local recurrence within the first two years after diagnosis. Therefore, delayed reconstruction when primary oncological management is completed, and the risk of local recurrence has reduced, should be considered.

Adjuvant radiotherapy has been demonstrated to improve local control, but not survival, in breast sarcomas and neoadjuvant radiotherapy does not have a role in this anatomical site [184, 185]. In borderline phyllodes tumours, surgical excision alone is likely to be curative, if negative margins are achieved [186]. Thus, adjuvant radiotherapy could be considered in high-risk cases of borderline phyllodes, such as large tumours and infiltrative margins, especially if clear margins could not be achieved surgically to improve local control. Malignant phyllodes tumours can often be large with an aggressive biology, therefore, consideration of adjuvant radiation treatment should be given in cases of large tumours (>5 cm), close (<5 mm) or positive margins, multifocal, or recurrent disease, irrespective of the surgery type (BCS versus mastectomy). In the case of close margins, repeat surgical excision to achieve clear margins, if possible, is preferred over adjuvant radiotherapy to optimise local control. As malignant phyllodes tumours have a strong association with TP53 mutations, testing for TP53 prior to offering adjuvant radiotherapy is advisable, especially in young patients, for assessment of secondary malignancy risks and patient counselling [187]. In high-grade primary sarcoma of the breast, adjuvant radiotherapy should be considered in cases of large tumours (>5 cm), close/positive margins, or recurrent disease. Radiation doses of 50–66 Gy in 1.8–2 Gy/fraction are generally recommended and hypo-fractionated schedules could be considered depending on institutional practice.

Radiation-induced sarcoma of the breast develop typically a number of years after therapeutic irradiation, usually following breast conserving surgery for epithelial breast cancer [20]. A sarcoma may develop as parenchymal radiation-induced breast sarcoma and/or cutaneous radiation-induced angiosarcoma. For radiation-induced breast sarcoma achieving widely clear surgical margins is especially important because further adjuvant radiotherapy is challenging to deliver, and only rarely offered, on a case by case basis. Surgery may mandate completion mastectomy with the inclusion of the underlying pectoralis major if the tumour abuts the muscle.

Angiosarcoma of the breast is a cutaneous malignancy with a particularly aggressive phenotype, a high risk of local relapse even in the context of negative margins, and a high likelihood of metastatic spread [20, 188]. Management of these tumours should be discussed at a specialist sarcoma MDT and surgery undertaken by a surgeon who is experienced in this disease. Close communication between a breast MDT and a sarcoma MDT is necessary to gauge the correct extent of surgery, the need for a resurfacing, reconstructive procedure to gain adequate margins [189], and the role of induction chemotherapy for locally advanced disease in which surgery may be morbid and occasionally oncologically.

**Table:** 

Key Recommendations : Breast Sarcomas
1) Close collaboration between a breast cancer MDT and a sarcoma MDT is necessary for the management of patients with breast sarcomas. Patients with sarcomatous differentiation within a metaplastic carcinoma should be managed as for an epithelial breast cancer.
2) For large, aggressive primary breast sarcomas or malignant phyllodes tumours breast conservation may not be possible, and reconstruction should be considered as a delayed rather than a synchronous procedure performed at the time of the mastectomy. Post operative radiotherapy should be considered for large and/or high-grade tumours with close /positive margins. (III,B)
3) Radiation induced angiosarcoma has an aggressive biology with a high risk of both local and distant relapse. Pre-operative communication between the breast and sarcoma MDT is of paramount importance. Surgery should be undertaken by a surgeon who is experienced in the management of this disease. Consideration should be given to resurfacing plastic surgical procedures to gain wide margins if necessary. Induction chemotherapy should be considered in locally advanced disease in which surgery would be excessively morbid and/or oncologically futile (IV,C)

### Skin sarcomas

Sarcomas arising in the skin or superficial subcutaneous tissues should be managed jointly between specialist skin cancer MDTs and sarcoma MDTs. However, many large subcutaneous sarcomas of a variety of histiotypes arise within subcutaneous tissues, often broadly abutting the deep fascia. The management of these sarcomas usually fits within a sarcoma MDT, whereas dermal sarcomas or sarcoma arising above the superficial fascia can be managed within a specialist skin cancer MDT.

#### Dermatofibrosarcoma protuberans (DFSP)

DFSP is a rare neoplasm of the dermal layer of the skin. Metastases are almost never seen in classic DFSP but may develop when the primary tumour has demonstrated fibrosarcomatous change. In this scenario the metastatic risk, as with any sarcoma will be determined by the size and grade of the fibrosarcoma component [190]. Classic DFSP has a tendency for local recurrence due to its infiltrative growth pattern, and potential for wide and deep extension from deep dermis into subcutaneous fat. Accordingly wide surgical margins have been advocated to account for that risk, although the precise extent of those margins is an area of debate [191–193]. Gaining negative histopathological margins is paramount for local control. Many tumours will present after an inadvertent surgical excision for a presumed benign abnormality with positive histological margins but no clinical evidence of residual disease. Under these circumstances it is acceptable for a conservative re-excision with primary closure to be undertaken, which in most circumstances will achieve negative margins and afford long-term control [194]. More radical resections with reconstruction can be reserved for the minority of patients in whom negative margins are not achieved. For DFSP arising at critical anatomical sites (face, distal extremities) where a wide excision would result in significant cosmetic deformity or functional loss, Mohs surgery can provide an alternative to initial wide excision and may be delivered through collaboration with a Skin Cancer MDT.

Radiotherapy should be considered for inoperable disease and can result in durable remissions. Adjuvant radiotherapy may also be used if the margins are involved, and re-excision is not possible [195].

Systemic treatment is appropriate in selected cases with unresectable or metastatic disease. DFSP is driven by a *t*(17;22) translocation that results in over-expression of platelet derived growth factor beta (PDGF*β*). Therefore, the PDGF*β* receptor may be inhibited by imatinib, which is licensed for the treatment of unresectable DFSP [196].

#### Atypical fibroxanthoma (AFX)/pleomorphic dermal sarcoma

AFX is a low-grade cutaneous spindle cell tumour considered a superficial variant of pleomorphic dermal sarcoma and can be histologically indistinguishable. It may be mistaken clinically or histologically for other spindle cell tumours. It is usually cured by surgical excision although local recurrence is common, but metastases are seen in less than 1% of cases. AFX with adverse pathological features - deep subcutis invasion, tumour necrosis, lympho-vascular invasion, or perineural invasion - may be regarded as pleomorphic dermal sarcomas [197]; they appear to share similar oncogene expression and mutations [198], but have a higher rate of metastases. Tumours which arise at sites where wide excision is not possible, for example commonly the scalp, may require adjuvant radiotherapy, and accordingly the reconstructive procedure will need to be planned to be robust to tolerate postoperative radiotherapy. Hence a preoperative core/punch biopsy to secure the diagnosis is favoured over a diagnostic excision biopsy and skin graft to resurface the defect. In PDS metastases are more often seen, potentially in around 16%, mainly to the lungs, and usually within 3 years of diagnosis [199], so at least a baseline CXR should be considered. Follow-up protocols may be influenced by comorbidity of patients, as many are elderly. Metastatic PDS can be treated in the same way as other soft tissue sarcomas, but as noted above there is some emerging evidence for the use of immune checkpoint inhibitors [141].

**Table:** 

Key Recommendations : Skin Sarcomas
1) Treatment of DFSP is wide surgical excision. In the case of an initial inadvertent enucleation of a DFSP, in the absence of macroscopic residual disease, a wide excision but with primary closure is an appropriate initial surgical strategy. Mohs surgery may be appropriate in selected cases to reduce functional loss at critical anatomical sites.(IV,C)
2) Adjuvant radiotherapy may be considered if surgical resection is incomplete, and re-excision not possible.(IV,A)
3) Imatinib may provide an option for neoadjuvant treatment in borderline resectable disease, or effective palliation for patients with unresectable DFSP
4) AFX is usually cured by surgical excision, although tumours with adverse pathological features are regarded as pleomorphic dermal sarcomas. Rarely metastases can occur.(IV,A)
5) Large subcutaneous pleomorphic dermal sarcomas should be diagnosed preoperatively by a core/ punch biopsy and surgery planned, taking into account the need for possible postoperative radiotherapy. (IV,B)

## Borderline tumours presenting to sarcoma MDTS

This group of soft tissue tumours are not considered typical sarcomas. They tend to remain localised, and whilst local recurrence following surgery can occur, they do not generally metastasise. They will almost always be referred to a sarcoma MDT for at least an opinion on treatment.

### Lipomas and atypical lipomatous tumours

The most common differential diagnosis seen by the sarcoma MDT is between lipoma and atypical lipomatous tumours (ALT), also known as well-differentiated liposarcoma (WDL). Essentially ALT and WDL are synonymous, as described in the WHO classification [7]. The latter term is more commonly applied to deep tumours in sites such as the retroperitoneum where surgical excision with a wide margin is unlikely, and therefore local recurrence is more common; progressive dedifferentiation with each recurrence is often observed. ALT/WDL of the extremities is distinct from lipoma in that it has the propensity for local recurrence, however dedifferentiation into a more aggressive disease is extremely rare.

MRI with expert review has been reported to differentiate between large, deep lipomas and ALT/WDL in up to 69% of cases [27, 200]. The variables of nodularity, septations, stranding, and relative size show an association with the diagnosis of ALT/well-differentiated liposarcoma [201, 202]. However, the defining diagnostic test to differentiate between lipomas and ALT/well differentiated liposarcoma is the molecular demonstration by fluorescence in-situ hybridisation of amplification of the MDM-2 cell cycle oncogene. This analysis can be done on percutaneous core needle biopsy if the diagnosis of ALT is suspected, and will alter the surgical approach [203].

Surgical resection is the usual treatment for ALT, and the prognosis is mostly excellent [204–206]. ALTs can be very large tumours abutting critical neurovascular structures and multiple muscle groups. However marginal resections as a complete en bloc specimen even if classified histopathologically as R1, will give excellent rates of long-term local control. In older patients, if surgery is likely to be morbid or the patient has significant comorbidities, radiological surveillance can be considered. In larger tumours, or those where clear margins are difficult to achieve, adjuvant radiotherapy may occasionally be considered [207]. Following post-operative wound care, patients with atypical lipomatous tumour can be discharged to primary care with re-referral only if there is clinical suspicion of a recurrence [208, 209].

**Table:** 

Key Recommendations : Lipomas and atypical lipomatous tumours :
1) Atypical lipomatous tumours of the extremities are biologically indolent tumours with a propensity for local relapse but little if any capacity for metastatic spread (IV)
2) Assessment by MRI cannot reliably differentiated between deep lipomas and ALTs and, in the case of diagnostic uncertainty, should be supplemented by percutaneous core needle biopsy to analyse for MDM-2 amplification (IV,B).
3) Complete *en bloc* resection, preserving adjacent neurovascular structures but with no attempt to gain wide surgical margins will afford long term local control (IV,B).

### Desmoid fibromatosis

Fibromatosis is a benign, clonal tumour which has a variable and unpredictable course but may be locally aggressive with a high symptom burden. Although usually sporadic it may occur in association with familial adenomatous polyposis (FAP) or Gardner syndrome, caused by germline mutations in the *APC* gene. Cases of sporadic fibromatosis usually harbour mutations in *CTNNB1*, the gene for beta-catenin. Young age, male gender, abdominal disease site, family history of bowel cancer, and absence of *CTNNB1* tumour mutation are risk factors for Gardner syndrome and should prompt investigation for germline *APC* mutation or colonoscopy. Given the consequences of missing this diagnosis it is recommended that *CTNNB1* mutation is part of the routine diagnostic workup.

The first step in management is active surveillance with regular MRI scans and clinical review. A comprehensive consensus document describing diagnosis and management was recently updated and provides a useful source of information [210].

Referral to dedicated pain service, physiotherapy, and psychological support should also be considered in parallel. Treatment choices may be influenced by anatomic site of disease and involvement of critical sites.

Standard treatment for progressing cases following an initial period of observation is medical therapy. The exception to this is fibromatosis arising in the abdominal wall, where relapse rates following surgery are low [211].

Choice of systemic treatment is considered in a step-wise manner starting with the least toxic treatment and incorporating individual patient considerations including fertility status. A placebo-controlled prospective randomised study has confirmed the activity of sorafenib but this is not routinely funded [212]. However, other systemic options exist, as discussed in the consensus documents, including pegylated liposomal doxorubicin, and pazopanib. Low dose chemotherapy including oral vinorelbine is well tolerated and has shown symptomatic benefit [213]. Nirogacestat, a γ-Secretase Inhibitor has also recently been shown in a phase 3 randomised placebo controlled trial to be beneficial in terms of progression free survival and symptom control (pain, physical function, and quality of life) [214].

Radiotherapy may be considered for patients with unresectable tumours in critical disease sites to achieve local control. Other local therapies such as cryoablation may be considered following multi-disciplinary discussion.

### Tenosynovial giant cell tumour (TGCT)

This benign but locally aggressive neoplastic disease presents as two forms as either a single nodule (localised, L-TGCT; previously GCT of tendon sheath or nodular tenosynovitis) or multiple nodules (diffuse-type, D-TGCT; previously pigmented villonodular synovitis, PVNS), generally affecting the synovium in young adults [7]. Recent international consensus guidelines have defined current best practise for management of these patients [215]. TGCT is usually treated by surgery alone, which is frequently curative for localised disease, but local relapses often occur after surgery for diffuse disease [216]. Arthroscopic or open synovectomy may have a role in diffuse disease, although increasingly it is now recognised that surgery may not be the optimal treatment for diffuse disease in view of the high rates of local recurrence and loss of function following surgery [215].

Radiotherapy is an effective treatment for TGCT, with high local control rates when given after surgery or as definitive treatment [217]. However, there are clear cautions about its use in the context of benign disease in a young population, with concerns of late radiation toxicity, and radiation-induced malignancy. For these reasons, radiotherapy is not considered a standard treatment for TGCT, particularly with the increasing role of effective systemic therapies [215]. However, there may be a role in a small number of very selected patients with symptomatic residual or recurrent disease for whom systemic therapy is not an option.

Due to a translocation involving the macrophage colony-stimulating factor (M-CSF or CSF1) gene seen in a proportion of cells, systemic therapies targeting CSF-1 can be used in TGCT. These are used in the context of symptomatic diffuse disease, to improve symptoms and function, although appropriate treatment endpoints optimal duration of treatment are unclear. Active agents include include small molecule inhibitors such as pexidatinib, imatinib, nilotinib, DCC-3014 (vimseltinib), and the monoclonal antibody RG7155 (emactuzumab) [218, 219], although the only licenced treatment (in USA only) is pexidartinib [219]. New targeted drugs are currently undergoing investigation.

**Table:** 

Key Recommendations: Desmoid fibromatosis and Tenosynovial giant cell tumour
1) A diagnosis of familial adenomatous polyposis (FAP) or Gardner syndrome needs to be considered in some fibromatosis cases. Initial standard treatment for fibromatosis is clinical and radiologic surveillance. (III,B)
2) Systemic treatments may be offered following initial surveillance for patients with symptomatic disease progression of fibromatosis. (III,B)
3) Radiotherapy and other local ablative therapies may be used for fibromatosis at critical sites.(IV,C)
4) Tenosynovial giant cell tumour (TGCT) may treated by surgery alone particularly for localised disease, although the role for surgery in diffuse disease is less clear. Radiotherapy is not a standard treatment, although may be indicated in very selected patients. (IV,C)
5) There is an increasing role for systemic therapies for diffuse symptomatic TGCT (I,B).

## References

[1] Grimer R, Judson I, Peake D, Seddon B. Guidelines for the management of soft tissue sarcomas. Sarcoma. 2010;2010:506182. 20634933 10.1155/2010/506182 PMC2903951

[2] Dangoor A, Seddon B, Gerrand C, Grimer R, Whelan J, Judson I. UK guidelines for the management of soft tissue sarcomas. Clin Sarcoma Res. 2016;6:1–26. 27891213 10.1186/s13569-016-0060-4 PMC5109663

[3] NHS England. Sarcoma Services (all ages) 2019 [Available from: https://www.england.nhs.uk/commissioning/publication/sarcoma-services-all-ages/.

[4] NICE. Improving outcomes for people with sarcoma 2006 [Available from: https://www.nice.org.uk/guidance/csg9.

[5] NICE. Sarcoma Service Specification 2015 [Available from: https://www.nice.org.uk/guidance/qs78.

[6] NICE. Improving outcomes in children and young people with cancer. Cancer service guideline CSG7. 2005.

[7] WHO. Classification of Tumours Editorial Board. Soft tissue and bone tumours. Lyon (France): International Agency for Research on Cancer. WHO Geneva, Switzerland; 2020. p. 104.

[8] Sarcoma UK. What is sarcoma? n.d. [Available from: https://sarcoma.org.uk/about-sarcoma/what-is-sarcoma/.

[9] Cancer Research UK. Bone sarcoma statistics n.d. [Available from: https://www.cancerresearchuk.org/health-professional/cancer-statistics/statistics-by-cancer-type/bone-sarcoma.

[10] Albritton K, Bleyer W. The management of cancer in the older adolescent. Eur J Cancer. 2003;39:2584–99. 14642921 10.1016/j.ejca.2003.09.013

[11] Birch J, Alston R, Quinn M, Kelsey A. Incidence of malignant disease by morphological type, in young persons aged 12–24 years in England, 1979–1997. Eur J Cancer. 2003;39:2622–31. 14642924 10.1016/j.ejca.2003.08.006

[12] Ferrari A, Bleyer A. Participation of adolescents with cancer in clinical trials. Cancer Treat Rev. 2007;33:603–8. 17250970 10.1016/j.ctrv.2006.11.005

[13] Kotilingam D, Lev DC, Lazar AJ, Pollock RE. Staging soft tissue sarcoma: evolution and change. CA: a cancer J Clin. 2006;56:282–91. 10.3322/canjclin.56.5.282 17005597

[14] Sarculator. Sarculator n.d. https://www.sarculator.com/.

[15] Rasmussen S, Friedman J. NF1 gene and neurofibromatosis 1. Am J Epidemiol. 2000;151:33–40. 10625171 10.1093/oxfordjournals.aje.a010118

[16] Uusitalo E, Rantanen M, Kallionpää RA, Pöyhönen M, Leppävirta J, Ylä-Outinen H, et al. Distinctive cancer associations in patients with neurofibromatosis type 1. J Clin Oncol. 2016;34:1978–86. 26926675 10.1200/JCO.2015.65.3576

[17] Chen CS, Suthers G, Carroll J, Rudzki Z, Muecke J. Sarcoma and familial retinoblastoma: clinical research. Clin Exp Ophthalmol. 2003;31:392–6. 14516425 10.1046/j.1442-9071.2003.00684.x

[18] Bell DW, Varley JM, Szydlo TE, Kang DH, Wahrer DC, Shannon KE, et al. Heterozygous germ line hCHK2 mutations in Li-Fraumeni syndrome. Science. 1999;286:2528–31. 10617473 10.1126/science.286.5449.2528

[19] Ballinger ML, Goode DL, Ray-Coquard I, James PA, Mitchell G, Niedermayr E, et al. Monogenic and polygenic determinants of sarcoma risk: an international genetic study. Lancet Oncol. 2016;17:1261–71. 27498913 10.1016/S1470-2045(16)30147-4

[20] Banks J, George J, Potter S, Gardiner M, Ives C, Shaaban A, et al. Breast Angiosarcoma Surveillance Study: UK national audit of management and outcomes of angiosarcoma of the breast and chest wall. Br J Surg. 2021;108:388–94. 33749771 10.1093/bjs/znaa128

[21] Neuhaus SJ, Pinnock N, Giblin V, Fisher C, Thway K, Thomas J, et al. Treatment and outcome of radiation-induced soft-tissue sarcomas at a specialist institution. Eur J Surg Oncol. 2009;35:654–9. 19112005 10.1016/j.ejso.2008.11.008

[22] Gladdy RA, Qin L-X, Moraco N, Edgar MA, Antonescu CR, Alektiar KM, et al. Do radiation-associated soft tissue sarcomas have the same prognosis as sporadic soft tissue sarcomas? J Clin Oncol. 2010;28:2064–9. 20308666 10.1200/JCO.2009.25.1728 PMC3651600

[23] Stewart FW, Treves N. Lymphangiosarcoma in postmastectomy lymphedema. A report of six cases in elephantiasis chirurgica. Cancer. 1948;1:64–81. 18867440 10.1002/1097-0142(194805)1:1<64::aid-cncr2820010105>3.0.co;2-w

[24] Smith G, Johnson G, Grimer R, Wilson S. Trends in presentation of bone and soft tissue sarcomas over 25 years: little evidence of earlier diagnosis. Ann R Coll Surg Engl. 2011;93:542–7. 22004638 10.1308/147870811X13137608455055 PMC3604925

[25] Lakkaraju A, Sinha R, Garikipati R, Edward S, Robinson P. Ultrasound for initial evaluation and triage of clinically suspicious soft-tissue masses. Clin Radiol. 2009;64:615–21. 19414084 10.1016/j.crad.2009.01.012

[26] NICE. Suspected cancer: recognition and referral, 2015. https://www.nice.org.uk/guidance/ng12.

[27] Cairncross L, Snow H, Strauss D, Smith M, Sjokvist O, Messiou C, et al. Diagnostic performance of MRI and histology in assessment of deep lipomatous tumours. J Br Surg. 2019;106:1794–9. 10.1002/bjs.11309 31502664

[28] BSG BSG. Ultrasound screening of soft tissue masses in the trunk and extremity: a British Sarcoma Group guide for ultrasonographers and primary care: BSG, British Sarcoma Group; 2019. https://britishsarcomagroup.org.uk/wp-content/uploads/2019/01/BSG-guidance-for-ultrasound-screening-of-soft-tissue-masses-in-the-trunk-and-extremity-FINAL-Jan-2019.pdf.

[29] King DM, Hackbarth DA, Kilian CM, Carrera GF. Soft-tissue sarcoma metastases identified on abdomen and pelvis CT imaging. Clin Orthop Relat Res®. 2009;467:2838–44. 19636646 10.1007/s11999-009-0989-1 PMC2758993

[30] Gronchi A, Miah A, Dei Tos A, Abecassis N, Bajpai J, Bauer S, et al. Soft tissue and visceral sarcomas: ESMO–EURACAN–GENTURIS Clinical Practice Guidelines for diagnosis, treatment and follow-up☆. Ann Oncol. 2021;32:1348–65. 34303806 10.1016/j.annonc.2021.07.006

[31] Stevenson JD, Watson JJ, Cool P, Cribb GL, Jenkins JP, Leahy M, et al. Whole-body magnetic resonance imaging in myxoid liposarcoma: A useful adjunct for the detection of extra-pulmonary metastatic disease. Eur J Surg Oncol. 2016;42:574–80. 26831007 10.1016/j.ejso.2015.12.011

[32] Roberge D, Vakilian S, Alabed YZ, Turcotte RE, Freeman CR, Hickeson M. FDG PET/CT in initial staging of adult soft-tissue sarcoma. Sarcoma. 2012;2012:960194. 23251096 10.1155/2012/960194 PMC3518058

[33] Federico SM, Spunt SL, Krasin MJ, Billup CA, Wu J, Shulkin B, et al. Comparison of PET–CT and conventional imaging in staging pediatric rhabdomyosarcoma. Pediatr Blood cancer. 2013;60:1128–34. 23255260 10.1002/pbc.24430 PMC4266929

[34] Quartuccio N, Fox J, Kuk D, Wexler LH, Baldari S, Cistaro A, et al. Pediatric bone sarcoma: diagnostic performance of 18F-FDG PET/CT versus conventional imaging for initial staging and follow-up. Am J Roentgenol. 2015;204:153–60. 25539251 10.2214/AJR.14.12932

[35] Ferner R, Golding JF, Smith M, Calonje E, Jan W, Sanjayanathan V, et al. [18F] 2-fluoro-2-deoxy-D-glucose positron emission tomography (FDG PET) as a diagnostic tool for neurofibromatosis 1 (NF1) associated malignant peripheral nerve sheath tumours (MPNSTs): a long-term clinical study. Ann Oncol. 2008;19:390–4. 17932395 10.1093/annonc/mdm450

[36] Brahmi M, Thiesse P, Ranchere D, Mognetti T, Pinson S, Renard C, et al. Diagnostic accuracy of PET/CT-guided percutaneous biopsies for malignant peripheral nerve sheath tumors in neurofibromatosis type 1 patients. PLoS One. 2015;10:e0138386. 26445379 10.1371/journal.pone.0138386 PMC4596851

[37] Van Houdt W, Schrijver A, Cohen-Hallaleh R, Memos N, Fotiadis N, Smith M, et al. Needle tract seeding following core biopsies in retroperitoneal sarcoma. Eur J Surg Oncol. 2017;43:1740–5. 28754227 10.1016/j.ejso.2017.06.009

[38] NHS. Rare and inherited disease eligibility criteria online2023 [5.2: https://www.england.nhs.uk/wp-content/uploads/2018/08/Rare-and-inherited-disease-eligibility-criteria-version-5.2.pdf.

[39] NHS. National genomic test directory for cancer online2023 [7.3: https://www.england.nhs.uk/wp-content/uploads/2018/08/cancer-national-genomic-test-directory-v7.3.xlsx.

[40] Thway K, Wang J, Mubako T, Fisher C. Histopathological diagnostic discrepancies in soft tissue tumours referred to a specialist centre: reassessment in the era of ancillary molecular diagnosis. Sarcoma. 2014;2014:686902. 25165418 10.1155/2014/686902 PMC4138733

[41] Ray-Coquard I, Montesco M, Coindre J, Dei Tos A, Lurkin A, Ranchère-Vince D, et al. Sarcoma: concordance between initial diagnosis and centralized expert review in a population-based study within three European regions. Ann Oncol. 2012;23:2442–9. 22331640 10.1093/annonc/mdr610 PMC3425368

[42] Guillou L, Coindre J-M, Bonichon F, Nguyen B, Terrier P, Collin F, et al. Comparative study of the National Cancer Institute and French Federation of Cancer Centers Sarcoma Group grading systems in a population of 410 adult patients with soft tissue sarcoma. J Clin Oncol. 1997;15:350–62. 8996162 10.1200/JCO.1997.15.1.350

[43] Trojani M, Contesso G, Coindre J, Rouesse J, Bui N, De Mascarel A, et al. Soft‐tissue sarcomas of adults; study of pathological prognostic variables and definition of a histopathological grading system. Int J cancer. 1984;33:37–42. 6693192 10.1002/ijc.2910330108

[44] Almond L, Tirotta F, Tattersall H, Hodson J, Cascella T, Barisella M, et al. Diagnostic accuracy of percutaneous biopsy in retroperitoneal sarcoma. J Br Surg. 2019;106:395–403. 10.1002/bjs.11064 30675910

[45] Marino-Enriquez A. Advances in the molecular analysis of soft tissue tumors and clinical implications. Surg Pathol Clin. 2015;8:525–37. 26297069 10.1016/j.path.2015.06.001 PMC4920057

[46] Amin MB, Edge SB, Greene FL, Byrd DR, Brookland RK, Washington MK, et al. AJCC cancer staging manual. Springer 2017.

[47] Callegaro D, Miceli R, Mariani L, Raut CP, Gronchi A. Soft tissue sarcoma nomograms and their incorporation into practice. Cancer. 2017;123:2802–20. 28493287 10.1002/cncr.30721

[48] van Praag VM, Rueten-Budde AJ, Jeys LM, Laitinen MK, Pollock R, Aston W, et al. A prediction model for treatment decisions in high-grade extremity soft-tissue sarcomas: personalised sarcoma care (PERSARC). Eur J Cancer. 2017;83:313–23. 28797949 10.1016/j.ejca.2017.06.032

[49] O’Donnell PW, Griffin AM, Eward WC, Sternheim A, Catton CN, Chung PW, et al. The effect of the setting of a positive surgical margin in soft tissue sarcoma. Cancer. 2014;120:2866–75. 24894656 10.1002/cncr.28793

[50] Le Grange F, Cassoni A, Seddon B. Tumour volume changes following pre-operative radiotherapy in borderline resectable limb and trunk soft tissue sarcoma. Eur J Surg Oncol. 2014;40:394–401. 24534361 10.1016/j.ejso.2014.01.011

[51] Roberge D, Skamene T, Nahal A, Turcotte RE, Powell T, Freeman C. Radiological and pathological response following pre-operative radiotherapy for soft-tissue sarcoma. Radiother Oncol. 2010;97:404–7. 21040989 10.1016/j.radonc.2010.10.007

[52] Fisher C, Thway K. Dataset for histopathological reporting of soft tissue sarcomas. 6 ed: Royal College of Pathologists; 2022.

[53] Enneking WF, Spanier SS, Goodman MA. A system for the surgical staging of musculoskeletal sarcoma. Clin Orthop Relat Res (1976–2007). 1980;153:106–20. 7449206

[54] Kainhofer V, Smolle M, Szkandera J, Liegl-Atzwanger B, Maurer-Ertl W, Gerger A, et al. The width of resection margins influences local recurrence in soft tissue sarcoma patients. Eur J Surg Oncol. 2016;42:899–906. 27107792 10.1016/j.ejso.2016.03.026

[55] Eggermont AM, de Wilt JH, ten Hagen TL. Current uses of isolated limb perfusion in the clinic and a model system for new strategies. Lancet Oncol. 2003;4:429–37. 12850194 10.1016/s1470-2045(03)01141-0

[56] Smith H, Cartwright J, Wilkinson M, Strauss D, Thomas J, Hayes A. Isolated limb perfusion with melphalan and tumour necrosis factor α for in-transit melanoma and soft tissue sarcoma. Ann Surg Oncol. 2015;22:356–61. 10.1245/s10434-015-4856-x 26350373

[57] Huis in ‘t Veld E, Grünhagen D, Verhoef C, Smith H, van Akkooi A, Jones R, et al. Isolated limb perfusion for locally advanced angiosarcoma in extremities: a multi-centre study. Eur J Cancer. 2017;85:114–21. 28918185 10.1016/j.ejca.2017.07.023

[58] Rosenberg SA, Tepper J, Glatstein E, Costa J, Baker A, Brennan M, et al. The treatment of soft-tissue sarcomas of the extremities: prospective randomized evaluations of (1) limb-sparing surgery plus radiation therapy compared with amputation and (2) the role of adjuvant chemotherapy. Ann Surg. 1982;196:305. 7114936 10.1097/00000658-198209000-00009 PMC1352604

[59] Yang JC, Chang AE, Baker AR, Sindelar WF, Danforth DN, Topalian SL, et al. Randomized prospective study of the benefit of adjuvant radiation therapy in the treatment of soft tissue sarcomas of the extremity. J Clin Oncol. 1998;16:197–203. 9440743 10.1200/JCO.1998.16.1.197

[60] Beane JD, Yang JC, White D, Steinberg SM, Rosenberg SA, Rudloff U. Efficacy of adjuvant radiation therapy in the treatment of soft tissue sarcoma of the extremity: 20-year follow-up of a randomized prospective trial. Ann Surg Oncol. 2014;21:2484–9. 24756814 10.1245/s10434-014-3732-4 PMC6293463

[61] Haas RL, DeLaney TF, O’Sullivan B, Keus RB, Le Pechoux C, Olmi P, et al. Radiotherapy for management of extremity soft tissue sarcomas: why, when, and where? Int J Radiat Oncol* Biol* Phys. 2012;84:572–80. 22520481 10.1016/j.ijrobp.2012.01.062

[62] O’Sullivan B, Davis AM, Turcotte R, Bell R, Catton C, Chabot P, et al. Preoperative versus postoperative radiotherapy in soft-tissue sarcoma of the limbs: a randomised trial. Lancet. 2002;359:2235–41. 12103287 10.1016/S0140-6736(02)09292-9

[63] Davis AM, O’Sullivan B, Turcotte R, Bell R, Catton C, Chabot P, et al. Late radiation morbidity following randomization to preoperative versus postoperative radiotherapy in extremity soft tissue sarcoma. Radiother Oncol. 2005;75:48–53. 15948265 10.1016/j.radonc.2004.12.020

[64] Robinson M, Gaunt P, Grimer R, Seddon B, Wylie J, Davis A, et al. Vortex trial: a randomized controlled multicenter phase 3 trial of volume of postoperative radiation therapy given to adult patients with extremity soft tissue sarcoma (STS). Int J Radiat Oncol Biol Phys. 2016;96:S1.

[65] VORTEX. Randomised trial of Volume of post-operative radiotherapy given to adult patients with eXtremity soft tissue sarcoma. University of Birmingham: University of Sheffield; 2010.

[66] Folkert MR, Singer S, Brennan MF, Kuk D, Qin L-X, Kobayashi WK, et al. Comparison of local recurrence with conventional and intensity-modulated radiation therapy for primary soft-tissue sarcomas of the extremity. J Clin Oncol. 2014;32:3236. 25185087 10.1200/JCO.2013.53.9452 PMC4178522

[67] Seddon B, Le Grange F, Simoes R, Stacey C, Shelly S, Sharon F, et al. Imris: a phase II study of intensity modulated radiotherapy (IMRT) in extremity soft tissue sarcoma (Sts). In Proceedigs of the connective tissue oncology society annual meeting; virtual 2020.

[68] Al Yami A, Griffin AM, Ferguson PC, Catton CN, Chung PW, Bell RS, et al. Positive surgical margins in soft tissue sarcoma treated with preoperative radiation: is a postoperative boost necessary? Int J Radiat Oncol* Biol* Phys. 2010;77:1191–7. 20056340 10.1016/j.ijrobp.2009.06.074

[69] Pan E, Goldberg SI, Chen YL, Giraud C, Hornick JL, Nielsen GP, et al. Role of post‐operative radiation boost for soft tissue sarcomas with positive margins following pre‐operative radiation and surgery. J Surg Oncol. 2014;110:817–22. 25111884 10.1002/jso.23741

[70] Koseła-Paterczyk H, Szacht M, Morysiński T, Ługowska I, Dziewirski W, Falkowski S, et al. Preoperative hypofractionated radiotherapy in the treatment of localized soft tissue sarcomas. Eur J Surg Oncol. 2014;40:1641–7. 25282099 10.1016/j.ejso.2014.05.016

[71] Parsai S, Lawrenz J, Kilpatrick S, Rubin B, Hymes C, Gray M, et al. Early outcomes of preoperative 5-fraction radiation therapy for soft tissue sarcoma followed by immediate surgical resection. Adv Radiat Oncol. 2020;5:1274–9. 33305088 10.1016/j.adro.2020.06.024 PMC7718495

[72] Kalbasi A, Kamrava M, Chu F-I, Telesca D, Van Dams R, Yang Y, et al. A phase II trial of 5-day neoadjuvant radiotherapy for patients with high-risk primary soft tissue sarcoma five-day neoadjuvant radiation for soft tissue sarcoma. Clin Cancer Res. 2020;26:1829–36. 32054730 10.1158/1078-0432.CCR-19-3524 PMC7189949

[73] Kepka L, DeLaney TF, Suit HD, Goldberg SI. Results of radiation therapy for unresected soft-tissue sarcomas. Int J Radiat Oncol* Biol* Phys. 2005;63:852–9. 16199316 10.1016/j.ijrobp.2005.03.004

[74] Slater JD, McNeese MD, Peters LJ. Radiation therapy for unresectable soft tissue sarcomas. Int J Radiat Oncol* Biol* Phys. 1986;12:1729–34. 3759524 10.1016/0360-3016(86)90312-3

[75] Allignet B, Waissi W, Geets X, Dufresne A, Brahmi M, Ray-Coquard I, et al. Long-term outcomes after definitive radiotherapy with modern techniques for unresectable soft tissue sarcoma. Radiother Oncol. 2022;173:55–61. 35640770 10.1016/j.radonc.2022.05.020

[76] RCR RCoR. Radiotherapy dose fractionation. 2019.

[77] DeLaney TF, Haas RL. Innovative radiotherapy of sarcoma: proton beam radiation. Eur J Cancer. 2016;62:112–23. 27258968 10.1016/j.ejca.2016.04.015

[78] Frisch S, Timmermann B. The evolving role of proton beam therapy for sarcomas. Clin Oncol. 2017;29:500–6. 10.1016/j.clon.2017.04.034 28506520

[79] NHS England. Proton beam therapy n.d. https://www.england.nhs.uk/commissioning/spec-services/highly-spec-services/pbt/.

[80] Salgado R, Van Marck E. Soft tissue tumours: the surgical pathologist’s perspective. In: De Schepper, A.M., Vanhoenacker, F., Gielen, J., Parizel, P.M. (eds) Imaging of soft tissue tumors. Springer, Berlin, Heidelberg; 2006. 10.1007/3-540-30792-3_8.

[81] Tierney J. Adjuvant chemotherapy for localised resectable soft-tissue sarcoma of adults: meta-analysis of individual data. Lancet. 1997;350:1647–54. 9400508

[82] Sarcoma Meta-Analysis Collaboration (SMAC). Adjuvant chemotherapy for localised resectable soft tissue sarcoma in adults. Cochrane Databases Syst Rev. 2000;CD001419. 10.1002/14651858.CD001419. 10.1002/14651858.CD001419 11034717

[83] Pervaiz N, Colterjohn N, Farrokhyar F, Tozer R, Figueredo A, Ghert M. A systematic meta‐analysis of randomized controlled trials of adjuvant chemotherapy for localized resectable soft‐tissue sarcoma. Cancer Interdiscip Int J Am Cancer Soc. 2008;113:573–81. 10.1002/cncr.23592 18521899

[84] Frustaci S, Gherlinzoni F, De Paoli A, Bonetti M, Azzarelli A, Comandone A, et al. Adjuvant chemotherapy for adult soft tissue sarcomas of the extremities and girdles: results of the Italian randomized cooperative trial. J Clin Oncol. 2001;19:1238–47. 11230464 10.1200/JCO.2001.19.5.1238

[85] Woll PJ, Reichardt P, Le Cesne A, Bonvalot S, Azzarelli A, Hoekstra HJ, et al. Adjuvant chemotherapy with doxorubicin, ifosfamide, and lenograstim for resected soft-tissue sarcoma (EORTC 62931): a multicentre randomised controlled trial. lancet Oncol. 2012;13:1045–54. 22954508 10.1016/S1470-2045(12)70346-7

[86] Callegaro D, Miceli R, Bonvalot S, Ferguson P, Strauss DC, Levy A, et al. Development and external validation of two nomograms to predict overall survival and occurrence of distant metastases in adults after surgical resection of localised soft-tissue sarcomas of the extremities: a retrospective analysis. Lancet Oncol. 2016;17:671–80. 27068860 10.1016/S1470-2045(16)00010-3

[87] Pasquali S, Pizzamiglio S, Touati N, Litiere S, Marreaud S, Kasper B, et al. The impact of chemotherapy on survival of patients with extremity and trunk wall soft tissue sarcoma: revisiting the results of the EORTC-STBSG 62931 randomised trial. Eur J Cancer. 2019;109:51–60. 30690293 10.1016/j.ejca.2018.12.009

[88] Pasquali S, Palmerini E, Quagliuolo V, Martin‐Broto J, Lopez‐Pousa A, Grignani G, et al. Neoadjuvant chemotherapy in high‐risk soft tissue sarcomas: a Sarculator‐based risk stratification analysis of the ISG‐STS 1001 randomized trial. Cancer. 2022;128:85–93. 34643947 10.1002/cncr.33895

[89] Palassini E, Ferrari S, Verderio P, De Paoli A, Martin Broto J, Quagliuolo V, et al. Feasibility of preoperative chemotherapy with or without radiation therapy in localized soft tissue sarcomas of limbs and superficial trunk in the Italian sarcoma Group/Grupo Espanol de Investigacion en Sarcomas Randomized Clinical Trial: three versus five cycles of full-dose epirubicin plus ifosfamide. J Clin Oncol. 2015;33:3628–34. 26351345 10.1200/JCO.2015.62.9394

[90] Grobmyer SR, Brennan MF. Predictive variables detailing the recurrence rate of soft tissue sarcomas. Curr Opin Oncol. 2003;15:319–26. 12874511 10.1097/00001622-200307000-00007

[91] MSK MSKCC. Prediction Tools n.d. https://www.mskcc.org/cancer-care/types/soft-tissue-sarcoma/prediction-tools.

[92] Toulmonde M, Le Cesne A, Mendiboure J, Blay JY, Piperno‐Neumann S, Chevreau C, et al. Long‐term recurrence of soft tissue sarcomas: prognostic factors and implications for prolonged follow‐up. Cancer. 2014;120:3003–6. 24942887 10.1002/cncr.28836

[93] Gerrand C, Billingham L, Woll P, Grimer R. Follow up after primary treatment of soft tissue sarcoma: a survey of current practice in the United Kingdom. Sarcoma. 2007;2007:34128. 18270541 10.1155/2007/34128 PMC2225460

[94] Puri A, Gulia A, Hawaldar R, Ranganathan P, Badwe RA. Does intensity of surveillance affect survival after surgery for sarcomas? Results of a randomized noninferiority trial. Clin Orthop Relat Res®. 2014;472:1568–75. 24249538 10.1007/s11999-013-3385-9 PMC3971232

[95] Rothermundt C, Whelan J, Dileo P, Strauss S, Coleman J, Briggs T, et al. What is the role of routine follow-up for localised limb soft tissue sarcomas? A retrospective analysis of 174 patients. Br J Cancer. 2014;110:2420–6. 24736584 10.1038/bjc.2014.200 PMC4021531

[96] Kwong TNK, Furtado S, Gerrand C. What do we know about survivorship after treatment for extremity sarcoma? A systematic review. Eur J Surg Oncol. 2014;40:1109–24. 24767804 10.1016/j.ejso.2014.03.015

[97] Blay JY, van Glabbeke M, Verweij J, Van Oosterom A, Le Cesne A, Oosterhuis J, et al. Advanced soft-tissue sarcoma: a disease that is potentially curable for a subset of patients treated with chemotherapy. Eur J Cancer. 2003;39:64–9. 12504660 10.1016/s0959-8049(02)00480-x

[98] Van Glabbeke M, Van Oosterom A, Oosterhuis J, Mouridsen H, Crowther D, Somers R, et al. Prognostic factors for the outcome of chemotherapy in advanced soft tissue sarcoma: an analysis of 2,185 patients treated with anthracycline-containing first-line regimens-a European Organization for Research and Treatment of Cancer Soft Tissue and Bone Sarcoma Group Study. J Clin Oncol. 1999;17:150–7. 10458228 10.1200/JCO.1999.17.1.150

[99] Judson I, Verweij J, Gelderblom H, Hartmann JT, Schöffski P, Blay J-Y, et al. Doxorubicin alone versus intensified doxorubicin plus ifosfamide for first-line treatment of advanced or metastatic soft-tissue sarcoma: a randomised controlled phase 3 trial. Lancet Oncol. 2014;15:415–23. 24618336 10.1016/S1470-2045(14)70063-4

[100] NIH NCI. SEER Stat Fact Sheets: Soft Tissue including Heart n.d.

[101] Harris SJ, Maruzzo M, Thway K, Al-Muderis O, Jones RL, Miah A, et al. Metastatic soft tissue sarcoma, an analysis of systemic therapy and impact on survival. JCO 2015;33:10545–10545. 10.1200/jco.2015.33.15_suppl.10545.

[102] Grünwald V, Litière S, Young R, Messiou C, Lia M, Wardelmann E, et al. Absence of progression, not extent of tumour shrinkage, defines prognosis in soft-tissue sarcoma–An analysis of the EORTC 62012 study of the EORTC STBSG. Eur J Cancer. 2016;64:44–51. 27323349 10.1016/j.ejca.2016.05.023

[103] Le Cesne A, Van Glabbeke M, Verweij J, Casali PG, Findlay M, Reichardt P, et al. Absence of progression as assessed by response evaluation criteria in solid tumors predicts survival in advanced GI stromal tumors treated with imatinib mesylate: the intergroup EORTC-ISG-AGITG phase III trial. J Clin Oncol. 2009;27:3969. 19620483 10.1200/JCO.2008.21.3330 PMC2799153

[104] NCCN NCCN. NCCN Guidelines n.d. https://www.nccn.org/guidelines/guidelines-detail?category=1&id=1464.

[105] Radaelli S, Stacchiotti S, Casali PG, Gronchi A. Emerging therapies for adult soft tissue sarcoma. Expert Rev Anticancer Ther. 2014;14:689–704. 24555529 10.1586/14737140.2014.885840

[106] Linch M, Miah AB, Thway K, Judson IR, Benson C. Systemic treatment of soft-tissue sarcoma—gold standard and novel therapies. Nat Rev Clin Oncol. 2014;11:187–202. 24642677 10.1038/nrclinonc.2014.26

[107] Noujaim J, Thway K, Sheri A, Keller C, Jones RL. Histology-driven therapy: the importance of diagnostic accuracy in guiding systemic therapy of soft tissue tumors. Int J Surg Pathol. 2016;24:5–15. 26399578 10.1177/1066896915606971

[108] Newton C, Ingram B. Ambulatory chemotherapy for teenagers and young adults. Br J Nurs. 2014;23:S36–S42. 24619053

[109] D’Ambrosio L, Touati N, Blay JY, Grignani G, Flippot R, Czarnecka AM, et al. Doxorubicin plus dacarbazine, doxorubicin plus ifosfamide, or doxorubicin alone as a first-line treatment for advanced leiomyosarcoma: a propensity score matching analysis from the European Organization for Research and Treatment of Cancer Soft Tissue and Bone Sarcoma Group. Cancer. 2020;126:2637–47. 32129883 10.1002/cncr.32795

[110] Van Oosterom A, Mouridsen H, Nielsen O, Dombernowsky P, Krzemieniecki K, Judson I, et al. Results of randomised studies of the EORTC Soft Tissue and Bone Sarcoma Group (STBSG) with two different ifosfamide regimens in first-and second-line chemotherapy in advanced soft tissue sarcoma patients. Eur J Cancer. 2002;38:2397–406. 12460784 10.1016/s0959-8049(02)00491-4

[111] Palumbo R, Palmeri S, Antimi M, Gatti C, Raffo P, Villani G, et al. Phase II study of continuous-infusion high-dose ifosfamide in advanced and/or metastatic pretreated soft tissue sarcomas. Ann Oncol. 1997;8:1159–62. 9426338 10.1023/a:1008279426654

[112] Buesa J, Lopez-Pousa A, Martin J, Anton A, del Muro JG, Bellmunt J, et al. Phase II trial of first-line high-dose ifosfamide in advanced soft tissue sarcomas of the adult: a study of the Spanish Group for Research on Sarcomas (GEIS). Ann Oncol. 1998;9:871–6. 9789610 10.1023/a:1008474802882

[113] Sleijfer S, Ouali M, van Glabbeke M, Krarup-Hansen A, Rodenhuis S, Le Cesne A, et al. Prognostic and predictive factors for outcome to first-line ifosfamide-containing chemotherapy for adult patients with advanced soft tissue sarcomas: an exploratory, retrospective analysis on large series from the European Organization for Research and Treatment of Cancer-Soft Tissue and Bone Sarcoma Group (EORTC-STBSG). Eur J Cancer. 2010;46:72–83. 19853437 10.1016/j.ejca.2009.09.022

[114] Martin-Liberal J, Alam S, Constantinidou A, Fisher C, Khabra K, Messiou C, et al. Clinical activity and tolerability of a 14-day infusional Ifosfamide schedule in soft-tissue sarcoma. Sarcoma. 2013;2013:868973. 24369450 10.1155/2013/868973 PMC3867825

[115] Leu KM, Ostruszka LJ, Shewach D, Zalupski M, Sondak V, Biermann JS, et al. Laboratory and clinical evidence of synergistic cytotoxicity of sequential treament with gemcitabine followed by docetaxel in the treatment of sarcoma. J Clin Oncol. 2004;22:1706–12. 15117993 10.1200/JCO.2004.08.043

[116] Maki RG, Wathen JK, Patel SR, Priebat DA, Okuno SH, Samuels B, et al. Randomized phase II study of gemcitabine and docetaxel compared with gemcitabine alone in patients with metastatic soft tissue sarcomas: results of sarcoma alliance for research through collaboration study 002 [corrected]. J Clin Oncol. 2007;25:2755–63. 17602081 10.1200/JCO.2006.10.4117

[117] Seddon B, Strauss SJ, Whelan J, Leahy M, Woll PJ, Cowie F, et al. Gemcitabine and docetaxel versus doxorubicin as first-line treatment in previously untreated advanced unresectable or metastatic soft-tissue sarcomas (GeDDiS): a randomised controlled phase 3 trial. Lancet Oncol. 2017;18:1397–1410. 10.1016/S1470-2045(17)30622-8. 10.1016/S1470-2045(17)30622-8 PMC5622179 28882536

[118] García-del-Muro X, López-Pousa A, Maurel J, Martín J, Martínez-Trufero J, Casado A, et al. Randomized phase II study comparing gemcitabine plus dacarbazine versus dacarbazine alone in patients with previously treated soft tissue sarcoma: a Spanish Group for Research on Sarcomas study. J Clin Oncol. 2011;29:2528–33. 21606430 10.1200/JCO.2010.33.6107

[119] Stacchiotti S, Tortoreto M, Bozzi F, Tamborini E, Morosi C, Messina A, et al. Dacarbazine in solitary fibrous tumor: a case series analysis and preclinical evidence vis-à-vis temozolomide and antiangiogenicsdacarbazine in solitary fibrous tumor. Clin Cancer Res. 2013;19:5192–201. 23888069 10.1158/1078-0432.CCR-13-0776

[120] Demetri GD, Chawla SP, von Mehren M, Ritch P, Baker LH, Blay JY, et al. Efficacy and safety of trabectedin in patients with advanced or metastatic liposarcoma or leiomyosarcoma after failure of prior anthracyclines and ifosfamide: results of a randomized phase II study of two different schedules. J Clin Oncol. 2009;27:4188–96. 19652065 10.1200/JCO.2008.21.0088

[121] Demetri GD, Von Mehren M, Jones RL, Hensley ML, Schuetze SM, Staddon A, et al. Efficacy and safety of trabectedin or dacarbazine for metastatic liposarcoma or leiomyosarcoma after failure of conventional chemotherapy: results of a phase III randomized multicenter clinical trial. J Clin Oncol. 2016;34:786. 26371143 10.1200/JCO.2015.62.4734 PMC5070559

[122] Le Cesne A, Cresta S, Maki RG, Blay JY, Verweij J, Poveda A, et al. A retrospective analysis of antitumour activity with trabectedin in translocation-related sarcomas. Eur J Cancer. 2012;48:3036–44. 22749255 10.1016/j.ejca.2012.05.012

[123] Kawai A, Araki N, Sugiura H, Ueda T, Yonemoto T, Takahashi M, et al. Trabectedin monotherapy after standard chemotherapy versus best supportive care in patients with advanced, translocation-related sarcoma: a randomised, open-label, phase 2 study. Lancet Oncol. 2015;16:406–16. 25795406 10.1016/S1470-2045(15)70098-7

[124] Skubitz KM, Haddad PA. Paclitaxel and pegylated‐liposomal doxorubicin are both active in angiosarcoma. Cancer Interdiscip Int J Am Cancer Soc. 2005;104:361–6. 10.1002/cncr.21140 15948172

[125] Barrett-Lee P, Dixon J, Farrell C, Jones A, Leonard R, Murray N, et al. Expert opinion on the use of anthracyclines in patients with advanced breast cancer at cardiac risk. Ann Oncol. 2009;20:816–27. 19153118 10.1093/annonc/mdn728

[126] Schlemmer M, Reichardt P, Verweij J, Hartmann J, Judson I, Thyss A, et al. Paclitaxel in patients with advanced angiosarcomas of soft tissue: a retrospective study of the EORTC soft tissue and bone sarcoma group. Eur J Cancer. 2008;44:2433–6. 18771914 10.1016/j.ejca.2008.07.037

[127] Mir O, Domont J, Cioffi A, Bonvalot S, Boulet B, Le Pechoux C, et al. Feasibility of metronomic oral cyclophosphamide plus prednisolone in elderly patients with inoperable or metastatic soft tissue sarcoma. Eur J Cancer. 2011;47:515–9. 21251814 10.1016/j.ejca.2010.11.025

[128] Schöffski P, Chawla S, Maki RG, Italiano A, Gelderblom H, Choy E, et al. Eribulin versus dacarbazine in previously treated patients with advanced liposarcoma or leiomyosarcoma: a randomised, open-label, multicentre, phase 3 trial. Lancet. 2016;387:1629–37. 26874885 10.1016/S0140-6736(15)01283-0

[129] Phillips E, Jones RL, Huang P, Digklia A. Efficacy of eribulin in soft tissue sarcomas. Front Pharmacol. 2022;13:869754. 35444542 10.3389/fphar.2022.869754 PMC9014307

[130] Van Der Graaf WT, Blay J-Y, Chawla SP, Kim D-W, Bui-Nguyen B, Casali PG, et al. Pazopanib for metastatic soft-tissue sarcoma (PALETTE): a randomised, double-blind, placebo-controlled phase 3 trial. Lancet. 2012;379:1879–86. 22595799 10.1016/S0140-6736(12)60651-5

[131] Frezza AM, Benson C, Judson IR, Litiere S, Marreaud S, Sleijfer S, et al. Pazopanib in advanced desmoplastic small round cell tumours: a multi-institutional experience. Clin Sarcoma Res. 2014;4:1–6. 25089183 10.1186/2045-3329-4-7 PMC4118147

[132] Maruzzo M, Martin-Liberal J, Messiou C, Miah A, Thway K, Alvarado R, et al. Pazopanib as first line treatment for solitary fibrous tumours: the Royal Marsden Hospital experience. Clin sarcoma Res. 2015;5:1–7. 10.1186/s13569-015-0022-2 PMC4320530 25664166

[133] Martin-Liberal J, Benson C, McCarty H, Thway K, Messiou C, Judson I. Pazopanib is an active treatment in desmoid tumour/aggressive fibromatosis. Clin Sarcoma Res. 2013;3:1–5. 24279994 10.1186/2045-3329-3-13 PMC4176486

[134] Rutkowski P, Klimczak A, Ługowska I, Jagielska B, Wągrodzki M, Dębiec-Rychter M, et al. Long-term results of treatment of advanced dermatofibrosarcoma protuberans (DFSP) with imatinib mesylate–the impact of fibrosarcomatous transformation. Eur J Surg Oncol. 2017;43:1134–41. 28365129 10.1016/j.ejso.2017.03.011

[135] Cassier PA, Gelderblom H, Stacchiotti S, Thomas D, Maki RG, Kroep JR, et al. Efficacy of imatinib mesylate for the treatment of locally advanced and/or metastatic tenosynovial giant cell tumor/pigmented villonodular synovitis. Cancer. 2012;118:1649–55. 21823110 10.1002/cncr.26409

[136] Demetri G, Antonescu CR, Bjerkehagen B, Bovee JV, Boye K, Chacón M, et al. Diagnosis and management of tropomyosin receptor kinase (TRK) fusion sarcomas: expert recommendations from the World Sarcoma Network. Ann Oncol. 2020;31:1506–17. 32891793 10.1016/j.annonc.2020.08.2232 PMC7985805

[137] Gounder M, Schöffski P, Jones RL, Agulnik M, Cote GM, Villalobos VM, et al. Tazemetostat in advanced epithelioid sarcoma with loss of INI1/SMARCB1: an international, open-label, phase 2 basket study. Lancet Oncol. 2020;21:1423–32. 33035459 10.1016/S1470-2045(20)30451-4

[138] Chen AP, Sharon E, O’Sullivan-Coyne G, Moore N, Foster JC, Hu JS, et al. Atezolizumab for advanced alveolar soft part sarcoma. N Engl J Med. 2023;389:911–21. 37672694 10.1056/NEJMoa2303383 PMC10729808

[139] Painter CA, Jain E, Tomson BN, Dunphy M, Stoddard RE, Thomas BS, et al. The Angiosarcoma Project: enabling genomic and clinical discoveries in a rare cancer through patient-partnered research. Nat Med. 2020;26:181–7. 32042194 10.1038/s41591-019-0749-z

[140] Martín-Broto J, Moura DS, Van Tine BA. Facts and hopes in immunotherapy of soft-tissue sarcomasfacts and hopes in immunotherapy of soft-tissue sarcomas. Clin Cancer Res. 2020;26:5801–8. 32601077 10.1158/1078-0432.CCR-19-3335 PMC7669707

[141] Helbig D, Klein S. Immune checkpoint inhibitors for unresectable or metastatic pleomorphic dermal sarcomas. Front Oncol. 2022;12:975342. 36465341 10.3389/fonc.2022.975342 PMC9712951

[142] Treasure T, Fiorentino F, Scarci M, Møller H, Utley M. Pulmonary metastasectomy for sarcoma: a systematic review of reported outcomes in the context of Thames Cancer Registry data. BMJ open. 2012;2:e001736. 23048062 10.1136/bmjopen-2012-001736 PMC3488730

[143] Campana LG, Bianchi G, Mocellin S, Valpione S, Campanacci L, Brunello A, et al. Electrochemotherapy treatment of locally advanced and metastatic soft tissue sarcomas: results of a non-comparative phase II study. World J Surg. 2014;38:813–22. 24170155 10.1007/s00268-013-2321-1

[144] Arthur A, Orton MR, Emsley R, Vit S, Kelly-Morland C, Strauss D, et al. A CT-based radiomics classification model for the prediction of histological type and tumour grade in retroperitoneal sarcoma (RADSARC-R): a retrospective multicohort analysis. Lancet Oncol. 2023;24:1277–86. 37922931 10.1016/S1470-2045(23)00462-X PMC10618402

[145] Messiou C, Morosi C. Imaging in retroperitoneal soft tissue sarcoma. J Surg Oncol. 2018;117:25–32. 29193092 10.1002/jso.24891 PMC5836919

[146] Swallow CJ, Strauss DC, Bonvalot S, Rutkowski P, Desai A, Gladdy RA, et al. Management of primary retroperitoneal sarcoma (RPS) in the adult: an updated consensus approach from the Transatlantic Australasian RPS Working Group. Ann Surg Oncol. 2021;28:7873–88. 33852100 10.1245/s10434-021-09654-z PMC9257997

[147] Thway K, Flora R, Shah C, Olmos D, Fisher C. Diagnostic utility of p16, CDK4, and MDM2 as an immunohistochemical panel in distinguishing well-differentiated and dedifferentiated liposarcomas from other adipocytic tumors. Am J Surg Pathol. 2012;36:462–9. 22301498 10.1097/PAS.0b013e3182417330

[148] Lee ATJ, Thway K, Huang PH, Jones RL. Clinical and molecular spectrum of liposarcoma. J Clin Oncol. 2018;36:151. 29220294 10.1200/JCO.2017.74.9598 PMC5759315

[149] Sirvent N, Coindre J-M, Maire G, Hostein I, Keslair F, Guillou L, et al. Detection of MDM2-CDK4 amplification by fluorescence in situ hybridization in 200 paraffin-embedded tumor samples: utility in diagnosing adipocytic lesions and comparison with immunohistochemistry and real-time PCR. Am J Surg Pathol. 2007;31:1476–89. 17895748 10.1097/PAS.0b013e3180581fff

[150] Berger-Richardson D, Burtenshaw SM, Ibrahim AM, Gladdy RA, Auer R, Beecroft R, et al. Early and late complications of percutaneous core needle biopsy of retroperitoneal tumors at two tertiary sarcoma centers. Ann Surg Oncol. 2019;26:4692–8. 31372868 10.1245/s10434-019-07656-6

[151] Gronchi A, Strauss DC, Miceli R, Bonvalot S, Swallow CJ, Hohenberger P, et al. Variability in patterns of recurrence after resection of primary retroperitoneal sarcoma (RPS). Ann Surg. 2016;263:1002–9. 26727100 10.1097/SLA.0000000000001447

[152] Dingley B, Fiore M, Gronchi A. Personalizing surgical margins in retroperitoneal sarcomas: an update. Expert Rev Anticancer Ther. 2019;19:613–31. 31159625 10.1080/14737140.2019.1625774

[153] Bonvalot S, Miceli R, Berselli M, Causeret S, Colombo C, Mariani L, et al. Aggressive surgery in retroperitoneal soft tissue sarcoma carried out at high-volume centers is safe and is associated with improved local control. Ann Surg Oncol. 2010;17:1507–14. 20393803 10.1245/s10434-010-1057-5

[154] Fairweather M, Gonzalez RJ, Strauss D, Raut CP. Current principles of surgery for retroperitoneal sarcomas. J Surg Oncol. 2018;117:33–41. 29315649 10.1002/jso.24919

[155] Pasquali S, Gronchi A, Strauss D, Bonvalot S, Jeys L, Stacchiotti S, et al. Resectable extra-pleural and extra-meningeal solitary fibrous tumours: a multi-centre prognostic study. Eur J Surg Oncol. 2016;42:1064–70. 26924782 10.1016/j.ejso.2016.01.023

[156] Bonvalot S, Gronchi A, Le Péchoux C, Swallow CJ, Strauss D, Meeus P, et al. Preoperative radiotherapy plus surgery versus surgery alone for patients with primary retroperitoneal sarcoma (EORTC-62092: STRASS): a multicentre, open-label, randomised, phase 3 trial. Lancet Oncol. 2020;21:1366–77. 32941794 10.1016/S1470-2045(20)30446-0

[157] STRASS 2. Surgery with or without neoadjuvant chemotherapy in high risk retroperitoneal sarcoma (STRASS2). European Organisation for Research and Treatment of Cancer (EORTC); 2019.

[158] Trans-Atlantic RPS Working Group. Management of recurrent retroperitoneal sarcoma (RPS) in the adult: a consensus approach from the Trans-Atlantic RPS Working Group. Ann Surg Oncol. 2016;23:3531–40. 27480354 10.1245/s10434-016-5336-7 PMC5557018

[159] RCOG. Morcellation for Myomectomy or Hysterectomy: RCOG Consent Advice No. 13. Royal College of Obstetricians and Gynaecologists; 2019.

[160] Park J-Y, Park S-K, Kim D-Y, Kim J-H, Kim Y-M, Kim Y-T, et al. The impact of tumor morcellation during surgery on the prognosis of patients with apparently early uterine leiomyosarcoma. Gynecol Oncol. 2011;122:255–9. 21565389 10.1016/j.ygyno.2011.04.021

[161] Morice P, Rodriguez A, Rey A, Pautier P, Atallah D, Genestie C, et al. Prognostic value of initial surgical procedure for patients with uterine sarcoma: analysis of 123 patients. Eur J Gynaecol Oncol. 2003;24:237–40. 12807231

[162] Seidman MA, Oduyebo T, Muto MG, Crum CP, Nucci MR, Quade BJ. Peritoneal dissemination complicating morcellation of uterine mesenchymal neoplasms. PloS one. 2012;7:e50058. 23189178 10.1371/journal.pone.0050058 PMC3506532

[163] Brohl AS, Li L, Andikyan V, Običan SG, Cioffi A, Hao K, et al. Age-stratified risk of unexpected uterine sarcoma following surgery for presumed benign leiomyoma. oncologist. 2015;20:433–9. 25765878 10.1634/theoncologist.2014-0361 PMC4391766

[164] USFDA UFaDA. UPDATED laparoscopic uterine power morcellation in hysterectomy and myomectomy: FDA Safety Communication. 2014.

[165] Seddon BM, Davda R. Uterine sarcomas—recent progress and future challenges. Eur J Radiol. 2011;78:30–40. 21247711 10.1016/j.ejrad.2010.12.057

[166] Reed N, Mangioni C, Malmström H, Scarfone G, Poveda A, Pecorelli S, et al. Phase III randomised study to evaluate the role of adjuvant pelvic radiotherapy in the treatment of uterine sarcomas stages I and II: an European Organisation for Research and Treatment of Cancer Gynaecological Cancer Group Study (protocol 55874). Eur J Cancer. 2008;44:808–18. European Organisation for Research and Treatment of Cancer Gynaecological Cancer Group 18378136 10.1016/j.ejca.2008.01.019

[167] Patel D, Handorf E, von Mehren M, Martin L, Movva S. Adjuvant chemotherapy in uterine leiomyosarcoma: Trends and factors impacting usage. Sarcoma. 2019;2019:3561501. 30881199 10.1155/2019/3561501 PMC6387708

[168] Chae SH, Shim S-H, Chang M, Choi AY, Kang GG, Lee SJ, et al. Effect of adjuvant therapy on the risk of recurrence in early-stage leiomyosarcoma: A meta-analysis. Gynecol Oncol. 2019;154:638–50. 31307664 10.1016/j.ygyno.2019.07.001

[169] Vaz J, Tian C, Richardson MT, Chan JK, Mysona D, Rao UN, et al. Impact of adjuvant treatment and prognostic factors in stage I uterine leiomyosarcoma patients treated in Commission on Cancer®-accredited facilities. Gynecol Oncol. 2020;157:121–30. 31954536 10.1016/j.ygyno.2019.12.008

[170] Hensley ML, Ishill N, Soslow R, Larkin J, Abu-Rustum N, Sabbatini P, et al. Adjuvant gemcitabine plus docetaxel for completely resected stages I–IV high grade uterine leiomyosarcoma: results of a prospective study. Gynecol Oncol. 2009;112:563–7. 19135708 10.1016/j.ygyno.2008.11.027

[171] Hensley ML, Wathen JK, Maki RG, Araujo DM, Sutton G, Priebat DA, et al. Adjuvant therapy for high‐grade, uterus‐limited leiomyosarcoma: results of a phase 2 trial (SARC 005). Cancer. 2013;119:1555–61. 23335221 10.1002/cncr.27942

[172] Samuels B, Chawla S, Patel S, Von Mehren M, Hamm J, Kaiser P, et al. Clinical outcomes and safety with trabectedin therapy in patients with advanced soft tissue sarcomas following failure of prior chemotherapy: results of a worldwide expanded access program study. Ann Oncol. 2013;24:1703–9. 23385197 10.1093/annonc/mds659

[173] Pautier P, Italiano A, Piperno-Neumann S, Chevreau C, Penel N, Firmin N, et al. Doxorubicin alone versus doxorubicin with trabectedin followed by trabectedin alone as first-line therapy for metastatic or unresectable leiomyosarcoma (LMS-04): a randomised, multicentre, open-label phase 3 trial. Lancet Oncol. 2022;23:1044–54. 35835135 10.1016/S1470-2045(22)00380-1

[174] George S, Feng Y, Manola J, Nucci MR, Butrynski JE, Morgan JA, et al. Phase 2 trial of aromatase inhibition with letrozole in patients with uterine leiomyosarcomas expressing estrogen and/or progesterone receptors. Cancer. 2014;120:738–43. 24222211 10.1002/cncr.28476

[175] Amant F, Floquet A, Friedlander M, Kristensen G, Mahner S, Nam EJ, et al. Gynecologic Cancer InterGroup (GCIG) consensus review for endometrial stromal sarcoma. Int J Gynecol Cancer. 2014;24:S67–72. 25033257 10.1097/IGC.0000000000000205

[176] Carroll A, Ramirez PT, Westin SN, Soliman PT, Munsell MF, Nick AM, et al. Uterine adenosarcoma: an analysis on management, outcomes, and risk factors for recurrence. Gynecol Oncol. 2014;135:455–61. 25449308 10.1016/j.ygyno.2014.10.022 PMC4430193

[177] Tanner EJ, Toussaint T, Leitao MM Jr., Hensley ML, Soslow RA, Gardner GJ, et al. Management of uterine adenosarcomas with and without sarcomatous overgrowth. Gynecol Oncol. 2013;129:140–4. 23283300 10.1016/j.ygyno.2012.12.036

[178] Schroeder BA, Rodler ET, Loggers ET, Pollack SM, Jones RL. Clinical benefit of trabectedin in uterine adenosarcoma. Med Oncol. 2013;30:501. 23456619 10.1007/s12032-013-0501-3

[179] Chu MC, Mor G, Lim C, Zheng W, Parkash V, Schwartz PE. Low-grade endometrial stromal sarcoma: hormonal aspects☆. Gynecol Oncol. 2003;90:170–6. 12821359 10.1016/s0090-8258(03)00258-0

[180] Lee C-H, Ou W-B, Mariño-Enriquez A, Zhu M, Mayeda M, Wang Y, et al. 14-3-3 fusion oncogenes in high-grade endometrial stromal sarcoma. Proc Natl Acad Sci. 2012;109:929–34. 22223660 10.1073/pnas.1115528109 PMC3271913

[181] Pencavel T, Allan CP, Thomas JM, Hayes A. Treatment for breast sarcoma: a large, single-centre series. Eur J Surg Oncol. 2011;37:703–8. 21628089 10.1016/j.ejso.2011.04.006

[182] Pencavel TD, Hayes A. Breast sarcoma–a review of diagnosis and management. Int J Surg. 2009;7:20–3. 19114317 10.1016/j.ijsu.2008.12.005

[183] Sars C, Sackey H, Frisell J, Dickman PW, Karlsson F, Kindts I, et al. Current clinical practice in the management of phyllodes tumors of the breast: an international cross-sectional study among surgeons and oncologists. Breast Cancer Res Treat. 2023;199:293–304. 36879102 10.1007/s10549-023-06896-1 PMC9988205

[184] Barth RJ Jr., Wells WA, Mitchell SE, Cole BF. A prospective, multi-institutional study of adjuvant radiotherapy after resection of malignant phyllodes tumors. Ann Surg Oncol. 2009;16:2288–94. 19424757 10.1245/s10434-009-0489-2 PMC5053421

[185] Chao X, Chen K, Zeng J, Bi Z, Guo M, Chen Y, et al. Adjuvant radiotherapy and chemotherapy for patients with breast phyllodes tumors: a systematic review and meta-analysis. BMC Cancer. 2019;19:372. 31014268 10.1186/s12885-019-5585-5 PMC6480723

[186] Choi N, Kim K, Shin KH, Kim Y, Moon H-G, Park W, et al. Malignant and borderline phyllodes tumors of the breast: a multicenter study of 362 patients (KROG 16-08). Breast Cancer Research and Treatment. 2018;171:335–44 29808288 10.1007/s10549-018-4838-3

[187] Frebourg T, Bajalica Lagercrantz S, Oliveira C, Magenheim R, Evans DG, European Reference Network G. Guidelines for the Li-Fraumeni and heritable TP53-related cancer syndromes. Eur J Hum Genet. 2020;28:1379–86. 32457520 10.1038/s41431-020-0638-4 PMC7609280

[188] Cohen-Hallaleh R, Smith H, Smith R, Stamp G, Al-Muderis O, Thway K, et al. Radiation induced angiosarcoma of the breast: outcomes from a retrospective case series. Clin Sarcoma Res. 2017;7:1–6. 28794852 10.1186/s13569-017-0081-7 PMC5547463

[189] Jallali N, James S, Searle A, Ghattaura A, Hayes A, Harris P. Surgical management of radiation-induced angiosarcoma after breast conservation therapy. Am J Surg. 2012;203:156–61. 21658671 10.1016/j.amjsurg.2010.12.011

[190] Huis in ‘t Veld EA, van Coevorden F, Grünhagen DJ, Smith MJ, van Akkooi AC, Wouters MW, et al. Outcome after surgical treatment of dermatofibrosarcoma protuberans: Is clinical follow‐up always indicated? Cancer. 2019;125:735–41. 30644528 10.1002/cncr.31924

[191] Huis in ‘t Veld E, Grünhagen D, Van Coevorden F, Smith M, van Akkooi A, Wouters M, et al. Adequate surgical margins for dermatofibrosarcoma protuberans—a multi-centre analysis. Eur J Surg Oncol. 2021;47:436–42. 32773140 10.1016/j.ejso.2020.06.022

[192] Chen Y, Jiang G. Association between surgical excision margins and outcomes in patients with dermatofibrosarcoma protuberans: a meta‐analysis. Dermatol Ther. 2021;34:e14954. 33835635 10.1111/dth.14954

[193] van Houdt WJ. Margins in DFSP reconsidered: Primum non nocere. Ann Surg Oncol. 2020;27:634–6. 31686341 10.1245/s10434-019-08012-4

[194] Snow H, Davies E, Strauss DC, Smith M, Hayes AJ. Conservative re-excision is a safe and simple alternative to radical resection in revision surgery for dermatofibrosarcoma protuberans. Ann Surg Oncol. 2020;27:919–23. 31664620 10.1245/s10434-019-08011-5

[195] Rutkowski P, Debiec-Rychter M. Current treatment options for dermatofibrosarcoma protuberans. Expert Rev Anticancer Ther. 2015;15:901–9. 26027711 10.1586/14737140.2015.1052799

[196] Rutkowski P, Dębiec‐Rychter M, Nowecki Z, Michej W, Symonides M, Ptaszynski K, et al. Treatment of advanced dermatofibrosarcoma protuberans with imatinib mesylate with or without surgical resection. J Eur Acad Dermatol Venereol. 2011;25:264–70. 20569296 10.1111/j.1468-3083.2010.03774.x

[197] Miller K, Goodlad JR, Brenn T. Pleomorphic dermal sarcoma: adverse histologic features predict aggressive behavior and allow distinction from atypical fibroxanthoma. Am J Surg Pathol. 2012;36:1317–26. 22510760 10.1097/PAS.0b013e31825359e1

[198] Helbig D, Ihle MA, Pütz K, Tantcheva-Poor I, Mauch C, Büttner R, et al. Oncogene and therapeutic target analyses in atypical fibroxanthomas and pleomorphic dermal sarcomas. Oncotarget. 2016;7:21763. 26943575 10.18632/oncotarget.7845 PMC5008321

[199] Orholt M, Abebe K, Rasmussen LE, Aaberg FL, Lindskov LJ, Schmidt G, et al. Atypical fibroxanthoma and pleomorphic dermal sarcoma: Local recurrence and metastasis in a nationwide population-based cohort of 1118 patients. J Am Acad Dermatol. 2023;89:1177–84. 37634740 10.1016/j.jaad.2023.08.050

[200] O’Donnell PW, Griffin AM, Eward WC, Sternheim A, White LM, Wunder JS, et al. Can experienced observers differentiate between lipoma and well-differentiated liposarcoma using only MRI? Sarcoma. 2013;2013:982784. 24385845 10.1155/2013/982784 PMC3872425

[201] Nagano S, Yokouchi M, Setoguchi T, Ishidou Y, Sasaki H, Shimada H, et al. Differentiation of lipoma and atypical lipomatous tumor by a scoring system: implication of increased vascularity on pathogenesis of liposarcoma. BMC Musculoskelet Disord. 2015;16:1–7. 25879189 10.1186/s12891-015-0491-8 PMC4340111

[202] Fisher SB, Baxter KJ, Staley CA III, Fisher KE, Monson DK, Murray DR, et al. The General Surgeon’s quandary: atypical lipomatous tumor vs lipoma, who needs a surgical oncologist? J Am Coll Surg. 2013;217:881–8. 24074812 10.1016/j.jamcollsurg.2013.06.003 PMC3805785

[203] Thway K, editor Well-differentiated liposarcoma and dedifferentiated liposarcoma: an updated review. Seminars in diagnostic pathology; 2019: Elsevier. 10.1053/j.semdp.2019.02.006 30852045

[204] Sommerville SM, Patton JT, Luscombe JC, Mangham DC, Grimer RJ. Clinical outcomes of deep atypical lipomas (well‐differentiated lipoma‐like liposarcomas) of the extremities. ANZ J Surg. 2005;75:803–6. 16173997 10.1111/j.1445-2197.2005.03519.x

[205] Kito M, Yoshimura Y, Isobe K, Aoki K, Momose T, Suzuki S, et al. Clinical outcome of deep-seated atypical lipomatous tumor of the extremities with median-term follow-up study. Eur J Surg Oncol. 2015;41:400–6. 25498358 10.1016/j.ejso.2014.11.044

[206] Mussi CE, Daolio P, Cimino M, Giardina F, De Sanctis R, Morenghi E, et al. Atypical lipomatous tumors: should they be treated like other sarcoma or not? Surgical consideration from a bi-institutional experience. Ann Surg Oncol. 2014;21:4090–7. 24962938 10.1245/s10434-014-3855-7

[207] Cassier P, Kantor G, Bonvalot S, Lavergne E, Stoeckle E, Le Péchoux C, et al. Adjuvant radiotherapy for extremity and trunk wall atypical lipomatous tumor/well-differentiated LPS (ALT/WD-LPS): a French Sarcoma Group (GSF-GETO) study. Ann Oncol. 2014;25:1854–60. 24914041 10.1093/annonc/mdu202

[208] Sivarajah G, Snow H, Wilkinson MJ, Strauss DC, Smith MJ, Hayes AJ. Low local recurrence rates following marginal surgical resection of non-coelomic Atypical Lipomatous Tumours/Well-differentiated Liposarcomas. Eur J Surg Oncol. 2023;50:107301. 38041960 10.1016/j.ejso.2023.107301

[209] Kalimuthu SN, Tilley C, Forbes G, Ye H, Lehovsky K, Pillay N, et al. Clinical outcome in patients with peripherally-sited atypical lipomatous tumours and dedifferentiated liposarcoma. J Pathol Clin Res. 2015;1:106–12. 27499897 10.1002/cjp2.12 PMC4858133

[210] DTWG DTWG. The management of desmoid tumours: a joint global consensus-based guideline approach for adult and paediatric patients. Eur J Cancer. 2020;127:96–107. 32004793 10.1016/j.ejca.2019.11.013

[211] Wilkinson MJ, Chan KE, Hayes AJ, Strauss DC. Surgical outcomes following resection for sporadic abdominal wall fibromatosis. Ann Surg Oncol. 2014;21:2144–9. 24604586 10.1245/s10434-014-3618-5

[212] Gounder MM, Mahoney MR, Van Tine BA, Ravi V, Attia S, Deshpande HA, et al. Sorafenib for advanced and refractory desmoid tumors. N. Engl J Med. 2018;379:2417–28. 30575484 10.1056/NEJMoa1805052 PMC6447029

[213] Gennatas S, Chamberlain F, Smrke A, Stewart J, Hayes A, Roden L, et al. A timely oral option: single‐agent vinorelbine in desmoid tumors. oncologist. 2020;25:e2013–e6. 32918789 10.1002/ONCO.13516 PMC8186406

[214] Gounder M, Ratan R, Alcindor T, Schoffski P, van der Graaf WT, Wilky BA, et al. Nirogacestat, a gamma-secretase inhibitor for desmoid tumors. N. Engl J Med. 2023;388:898–912. 36884323 10.1056/NEJMoa2210140 PMC11225596

[215] Stacchiotti S, Dürr HR, Schaefer I-M, Woertler K, Haas R, Trama A, et al. Best clinical management of tenosynovial giant cell tumour (TGCT): a consensus paper from the community of experts. Cancer Treat Rev. 2023;112:102491. 36502615 10.1016/j.ctrv.2022.102491

[216] Palmerini E, Staals EL, Maki RG, Pengo S, Cioffi A, Gambarotti M, et al. Tenosynovial giant cell tumour/pigmented villonodular synovitis: outcome of 294 patients before the era of kinase inhibitors. Eur J Cancer. 2015;51:210–7. 25465190 10.1016/j.ejca.2014.11.001

[217] Griffin AM, Ferguson PC, Catton CN, Chung PW, White LM, Wunder JS, et al. Long‐term outcome of the treatment of high‐risk tenosynovial giant cell tumor/pigmented villonodular synovitis with radiotherapy and surgery. Cancer. 2012;118:4901–9. 22281719 10.1002/cncr.26529

[218] Palmerini E, Staals EL. Treatment updates on tenosynovial giant cell tumor. Curr Opin Oncol. 2022;34:322–7. 35837703 10.1097/CCO.0000000000000853

[219] Tap WD, Gelderblom H, Palmerini E, Desai J, Bauer S, Blay JY, et al. Pexidartinib versus placebo for advanced tenosynovial giant cell tumour (ENLIVEN): a randomised phase 3 trial. Lancet. 2019;394:478–87. 31229240 10.1016/S0140-6736(19)30764-0 PMC6860022

[220] Rothermundt C, Andreou D, Blay J-Y, Brodowicz T, Desar IM, Dileo P, et al. Controversies in the management of patients with soft tissue sarcoma: recommendations of the conference on state of science in sarcoma 2022. Eur J Cancer. 2023;180:158–79. 36599184 10.1016/j.ejca.2022.11.008

[221] Stacchiotti S, Ferrari S, Redondo A, Hindi N, Palmerini E, Salgado MAV, et al. Pazopanib for treatment of advanced extraskeletal myxoid chondrosarcoma: a multicentre, single-arm, phase 2 trial. Lancet Oncol. 2019;20:1252–62. 31331701 10.1016/S1470-2045(19)30319-5

[222] Stacchiotti S, Baldi GG, Morosi C, Gronchi A, Maestro R. Extraskeletal myxoid chondrosarcoma: state of the art and current research on biology and clinical management. Cancers. 2020;12:2703. 32967265 10.3390/cancers12092703 PMC7563993

[223] Martin-Broto J, Mondaza-Hernandez JL, Moura DS, Hindi N. A comprehensive review on solitary fibrous tumor: new insights for new horizons. Cancers. 2021;13:2913. 34200924 10.3390/cancers13122913 PMC8230482

[224] Toulmonde M, Pulido M, Ray-Coquard I, André T, Isambert N, Chevreau C, et al. Pazopanib or methotrexate–vinblastine combination chemotherapy in adult patients with progressive desmoid tumours (DESMOPAZ): a non-comparative, randomised, open-label, multicentre, phase 2 study. Lancet Oncol. 2019;20:1263–72. 31331699 10.1016/S1470-2045(19)30276-1

[225] Stacchiotti S, Negri T, Zaffaroni N, Palassini E, Morosi C, Brich S, et al. Sunitinib in advanced alveolar soft part sarcoma: evidence of a direct antitumor effect. Ann Oncol. 2011;22:1682–90. 21242589 10.1093/annonc/mdq644

[226] Judson I, Morden JP, Kilburn L, Leahy M, Benson C, Bhadri V, et al. Cediranib in patients with alveolar soft-part sarcoma (CASPS): a double-blind, placebo-controlled, randomised, phase 2 trial. Lancet Oncol. 2019;20:1023–34. 31160249 10.1016/S1470-2045(19)30215-3 PMC6602919

[227] Naqash AR, Coyne GHOS, Moore N, Sharon E, Takebe N, Fino KK, et al. Phase II study of atezolizumab in advanced alveolar soft part sarcoma (ASPS). JCO 2021;39: 11519–11519. 10.1200/JCO.2021.39.15_suppl.11519.

[228] Wilky BA, Trucco MM, Subhawong TK, Florou V, Park W, Kwon D, et al. Axitinib plus pembrolizumab in patients with advanced sarcomas including alveolar soft-part sarcoma: a single-centre, single-arm, phase 2 trial. Lancet Oncol. 2019;20:837–48. 31078463 10.1016/S1470-2045(19)30153-6

[229] Martin-Broto J, Hindi N, Grignani G, Martinez-Trufero J, Redondo A, Valverde C, et al. Nivolumab and sunitinib combination in advanced soft tissue sarcomas: a multicenter, single-arm, phase Ib/II trial. J Immunother cancer. 2020;8:e001561. 33203665 10.1136/jitc-2020-001561 PMC7674086

[230] Stacchiotti S, Miah A, Frezza A, Messiou C, Morosi C, Caraceni A, et al. Epithelioid hemangioendothelioma, an ultra-rare cancer: a consensus paper from the community of experts. ESMO Open. 2021;6:100170. 34090171 10.1016/j.esmoop.2021.100170 PMC8182432

[231] Schöffski P, Sufliarsky J, Gelderblom H, Blay J-Y, Strauss SJ, Stacchiotti S, et al. Crizotinib in patients with advanced, inoperable inflammatory myofibroblastic tumours with and without anaplastic lymphoma kinase gene alterations (European Organisation for Research and Treatment of Cancer 90101 CREATE): a multicentre, single-drug, prospective, non-randomised phase 2 trial. Lancet Respir Med. 2018;6:431–41. 29669701 10.1016/S2213-2600(18)30116-4

[232] Wagner AJ, Malinowska-Kolodziej I, Morgan JA, Qin W, Fletcher CD, Vena N, et al. Clinical activity of mTOR inhibition with sirolimus in malignant perivascular epithelioid cell tumors: targeting the pathogenic activation of mTORC1 in tumors. J Clin Oncol. 2010;28:835. 20048174 10.1200/JCO.2009.25.2981 PMC4810029

[233] Wagner AJ, Ravi V, Riedel RF, Ganjoo K, Van Tine BA, Chugh R, et al. nab-Sirolimus for patients with malignant perivascular epithelioid cell tumors. J Clin Oncol. 2021;39:3660–70. 34637337 10.1200/JCO.21.01728 PMC8601264

